# Artificial intelligence and neoantigens: paving the path for precision cancer immunotherapy

**DOI:** 10.3389/fimmu.2024.1394003

**Published:** 2024-05-29

**Authors:** Alla Bulashevska, Zsófia Nacsa, Franziska Lang, Markus Braun, Martin Machyna, Mustafa Diken, Liam Childs, Renate König

**Affiliations:** ^1^ Host-Pathogen-Interactions, Paul-Ehrlich-Institut, Langen, Germany; ^2^ TRON - Translational Oncology at the University Medical Center of the Johannes Gutenberg University gGmbH, Mainz, Germany

**Keywords:** neoantigen prediction, artificial intelligence, immunopeptidomics, cancer immunotherapy, precision medicine

## Abstract

Cancer immunotherapy has witnessed rapid advancement in recent years, with a particular focus on neoantigens as promising targets for personalized treatments. The convergence of immunogenomics, bioinformatics, and artificial intelligence (AI) has propelled the development of innovative neoantigen discovery tools and pipelines. These tools have revolutionized our ability to identify tumor-specific antigens, providing the foundation for precision cancer immunotherapy. AI-driven algorithms can process extensive amounts of data, identify patterns, and make predictions that were once challenging to achieve. However, the integration of AI comes with its own set of challenges, leaving space for further research. With particular focus on the computational approaches, in this article we have explored the current landscape of neoantigen prediction, the fundamental concepts behind, the challenges and their potential solutions providing a comprehensive overview of this rapidly evolving field.

## 1 Introduction

Recently, there has been an increasing number of reports on promising treatment paradigms based on reactivation of the immune system against cancer cells. Cancer immunotherapies aim to counteract the tactics employed by tumors that deactivate the immune system. Nevertheless, solely reactivating the immune system is not enough for the thorough elimination of tumors. It is essential that the reactivated immune system can distinguish malignant cells from their healthy counterparts.

The immune recognition of tumor tissues primarily relies on tumor antigens. Short antigenic peptides derived from tumor antigens are presented on the surface of the tumor cell by major histocompatibility complex (MHC) molecules serving as targets for the antitumor immune response. In humans, the MHC-I and MHC-II proteins are encoded by Human Leukocyte Antigen (HLA) genes, which are polymorphic in the human population. Given that the tumor antigens are the major target for antitumor T cells, they play a pivotal role in effective tumor elimination. Tumor antigens are typically categorized as tumor-associated antigens (TAA) and tumor-specific antigens (TSA). TAAs include antigens derived from genes overexpressed in cancer cells due to their malignant transformation, and comprise a class of normal self-proteins that are minimally expressed by healthy tissues. TAAs are generally weakly immunogenic due to central immune tolerance mechanisms. In contrast, TSAs are expressed exclusively on tumor cells. Most TSAs are neoantigens resulting from somatic mutations, such as insertion or deletions (INDELs), single nucleotide variants (SNVs), frameshifts and gene fusions (1). Since these neoantigens are products of tumor-specific irregularities, they are less susceptible to central immune tolerance, making them suitable candidates for therapeutic targeting.

Neoantigen cancer vaccines have emerged as a novel clinical approach to treat cancer (2). The purpose of a personalized anticancer vaccine is to direct T cells towards tumor eradication by leveraging neoantigens while preserving healthy tissue. There are two broad categories of immunotherapy treatments. Vaccinating against cancer induces long-lasting *de novo* antitumor immunity and is termed active immunotherapy (3, 4). Adoptive cell therapy (ACT) approaches, such as adoptive transfer of tumor-infiltrating lymphocytes (TILs), transgenic T cells, or chimeric antigen receptor T cells are based on the *in vitro* generation of tumor-specific T cells with subsequent infusion to the patient (passive immunotherapy). Currently, there is a variety of clinical trials, testing neoantigen-based anticancer vaccines either independently or in conjunction with other immunotherapies, checkpoint inhibitors or novel drugs under investigation. Numerous articles comprehensively review the field of mutation-derived neoantigen cancer vaccines. For detailed insights into preclinical and clinical studies, we recommend the review of Aurisicchio et al. (5). The review paper of Shemesh et al. (6) presents the clinical trial landscape of personalized therapeutic cancer vaccines, highlighting their opportunities and emerging challenges. Further insights into the challenges associated with targeting cancer neoantigens are outlined in the work of Chen et al. (7). Designing neoantigen cancer vaccines, trials, and trial outcomes are described in Biswas et al.’s work (8).

Detection of neoantigens is crucial for developing personalized cancer immunotherapies. Currently artificial intelligence (AI) is widely used to assess the factors that shape tumor immunogenicity. The use of AI for neoantigen prediction enhances the accuracy, efficiency, and personalized nature of cancer immunotherapy development by effectively analyzing and interpreting complex genomic data. However, the identification of putative neoantigens from genomic data still remains a challenge. To address this, specialized software tools have been developed for specific sub-tasks such as HLA typing and in silico prediction of peptide binding affinity to MHC molecules. Complex pipelines that encompass multiple analytical tasks have also been created. Current strategies for the identification of neoantigens are extensively reviewed in multiple articles (9–11).

**Figure 1:**
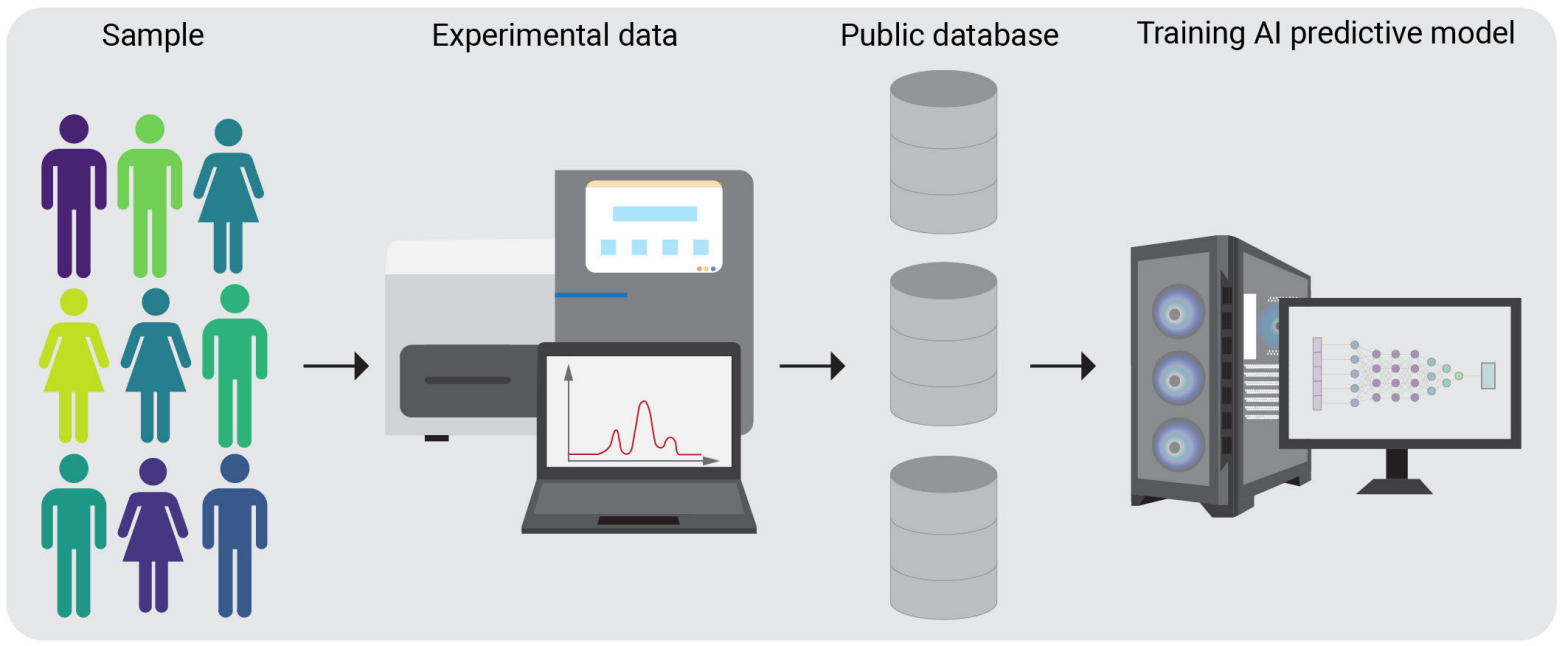
Schematic overview of AI algorithm training on public databases. A group of subjects, specific for the condition of interest is chosen for the experimental procedures. After completing the experimental pipelines, the generated data is stored in a public database. AI algorithms can then be trained on these datasets.

For the successful implementation of AI vast amount of data is required. Genomic data comes in various forms, such as DNA sequences, RNA expression profiles. AI models can be trained to handle diverse data types, allowing for a more comprehensive, fast analysis of the factors influencing neoantigen formation. Significant amounts of high-throughput biomedical data, including omics and immunological data, have been accumulated in public databases, and can be transformed into novel insights. These data can be used for training a model with AI - based computational algorithm to properly interpret the data and learn from it in order to make accurate decisions based on the input information provided (**Figure 1**). Additionally, AI models can help to identify novel neoantigens by recognizing patterns and associations in the molecular and cellular profiling data that may be challenging with the traditional methods.

Most state-of-the-art computational approaches for ranking and selecting candidate neoantigens predominantly rely on prediction methods, rooted in conventional machine learning (ML) algorithms, including artificial neural networks (ANNs), and modern AI architectures, trained on large experimental datasets.

Artificial Neural Networks are computational models inspired by biological neural networks. They learn the relationship between the inputs and outputs using samples from the training dataset (e.g., peptide sequences) and make predictions for the new samples. ANN’s are optimized by adjusting their parameters (weights and biases) based on the difference between the predicted values and actual values, utilizing the error-correction-learning rule known as *back propagation.*

Deep Learning (DL), a subset of machine learning and artificial intelligence stemming from ANNs, has gained increasing attention over the past years. The most commonly applied architectures include deep neural networks (DNNs) and convolutional neural networks (CNNs). DNNs consist of an input layer, multiple hidden layers, and an output layer with nodes in adjacent layers fully interconnected. CNNs primarily feature convolutional and pooling layers, often followed by fully connected layers. For an in-depth understanding of deep learning principles and concepts, we recommend the book of Goodfellow et al. (12). For definitions of AI and DL-related terms, please refer to our AI glossary (**Table 1**).

**Table 1:** – AI glossary.

Terms	Definitions
**Artificial Intelligence (AI)**	Field of computer science developing approaches possessing intelligent capabilities for learning, reasoning, planning, prediction, problem-solving and decision making.
**Artificial Neural Network (ANN)**	Models of computation inspired by human brain and consisting of a collection of interconnected neurons.
**Attention module**	Assigns weights to individual parts of the input and learns to assign higher weights, attention values, to those inputs that make a greater contribution to the prediction.
**Back propagation**	Algorithm used for training of ANN i.e. updating its parameters by applying the chain rule of differentiation starting from the network output and propagating the gradients backward.
**Bidirectional Encoder Representations from Transformers (BERT)**	A large scale model pre-trained on large amounts of unannotated data, which can be fine-tuned to the final model using another smaller task-specific dataset.
**Bidirectional Recurrent Neural Network (BiRNN)**	Labels each element of the input sequence based on the element’s past and future contexts by concatenating the outputs of two RNNs, one processing the sequence from left to right, the other one from right to left.
**Binary classification**	Classification task where each input sample should be categorized into two exclusive categories.
**Capsule Neural Network (CapsNet)**	Type of ANN attempting to better model hierarchical relationships and mimic biological neural organization more closely.
**Convolutional Neural Network (CNN)**	Employs convolutional layers which function as feature detectors learning filters (sets of weights) applied to all parts of the input in parallel.
**Deep Learning (DL)**	Type of ML imitating the way how brain gains knowledge, employing highly nonlinear neural network models to learn representations or features of the data for the prediction task at hand.
**Embedding**	Multidimensional numeric vector or intermediate CNN output which can be considered as encoding or representation of the input data.
**Ensemble Learning**	Technique to combine multiple machine learning algorithms to generate more accurate prediction than a single model.
**Explainable AI/Explainability**	AI approaches having the goal to make decision logic and reasoning of AI algorithms trusted and easily understood by humans.
**Fine-tuning**	Additional training of existing, pre-trained model on a new context- or task- specific data.
**Gated Recurrent Unit (GRU)**	Variation of LSTM without memory unit. Works better for smaller datasets.
**Generalization**	refers to how well the trained model performs on data it has never seen before.
**Generative Pre-trained Transformer (GPT)**	Large language model (*LLM*) developed by OpenAI. LLMs can have billions of parameters.
**Learning or Optimization**	the process of adjusting a model to get the best performance possible on the training data.
**Long Short-Term Memory (LSTM)**	Evolution of RNN capable to learn which information from the past (previous words of the sentence) should be used for the current output and which can simply be forgotten.
**Machine Learning (ML)**	Process of construction a model based on sample data or experience, known as **training data**, capable to make predictions or decisions about the future previously unseen samples.
**Multiple Instance Learning**	Learning paradigm which allows the training of a classifier from ambiguously labeled data. In particular, rather than providing the learning algorithm with input/label pairs, labels are assigned to sets or bags of inputs.
**Natural Language Processing (NLP)**	Subfield of AI focusing on the ability of computers to read and analyze large volumes of unstructured language data (e.g., text).
**Neuron (Perceptron)**	Computational unit. Computes a weighted sum of its inputs and applies a nonlinear activation function to calculate its output.
**Overfitting**	Occurs when a model learned patterns that are specific to the training data but irrelevant when it comes to new data.
**Parameters**	A set of numerical values in an AI model (e.g. weights of neural connections in ANN) that are determined by training.
**Recurrent Neural Network (RNN)**	Type of ANN introduced for sequential data processing. Each node in the RNN functions as a memory cell, in which the output is transmitted back to the RNN neuron rather than only passing it to the next node.
**Self-supervised Learning**	supervised learning without human-annotated labels. The labels are still involved but they’re generated from the input data.
**Supervised Learning**	Consists of learning to map input data to known targets (also called *annotations*), given a set of examples (often annotated by humans).
**Transfer Learning**	The process of using pre-trained model and quickly retrain it for the new task, or add additional layers on top, rather than training a new model from scratch.
**Transformer**	NLP model trained on a large data set of sentences for the task of inferring missing words that fit both in terms of grammar and semantics taking into account the surrounding context.
**Unsupervised Learning**	Finding interesting patterns or transformations of the input data without the help of any annotations.

Notable applications of deep learning in biomedicine, including medical imaging and drug discovery, are comprehensively covered in Wainberg et al. (13), while Wen et al. (14) delve into DL methods in proteomics.

Deep learning requires all input and output variables to be numeric. One important aspect of DL is data preprocessing or input encoding, which transforms raw data, such as peptide or protein sequences, into a suitable format for learning. Designing novel representation methods for protein sequence data is an active research direction. For example, the DeepLigand (15) approach treats each peptide sequence as a sentence, and each amino acid as a word, using the deep language model ELMo (16) to embed peptides into vector representations for tasks like peptide-MHC binding affinity prediction.

In addition to DNN and CNN, other DL architectures, such as gated recurrent unit (GRU) and long short-term memory (LSTM) neural networks, have proven effective for the peptide sequence-based prediction tasks. These methods can model dependences between amino acid residues within peptides of varying lengths without artificial lengthening or shortening, and they tend to be substantially faster than standard neural networks.

Recent advances in Natural Language Processing (NLP) have demonstrated the effectiveness of complex models, such as *Transformers*, including BERT (Bidirectional Encoder Representations from Transformers) (17), and GPT (Generative Pretrained Transformer) (Radford et al., 2018)^1^, in learning rich contextual word representations. They can be trained to understand semantics from text without labels *(self-supervised learning)* (18). Similar techniques have also been applied to learn features from a large corpus of protein sequence data from public datasets (19, 20).

Another important characteristic of DL is *transfer learning*, which involves initializing training with representations learned from a previous task. Instead of training a new network from scratch, pretrained models can be downloaded and further trained for a new task by adding additional layers or *fine-tuned* using the new data. Examples include BERTMHC (21), MHCRoBERTa (22) which use transformers and transfer learning for peptide-MHC binding prediction. The authors found that leveraging self-supervised pretraining on large protein sequence corpora can lead to improved performance, particularly when training data is limited.

Achieving optimal prediction accuracy requires the tuning of model settings, or *hyperparameters*, e.g. determining how fast the weights of NN should be adjusted during training. Hyperparameter search techniques use validation examples that are held out from training. We provide the reader with a helpful background for understanding approaches assessing the performance of AI systems and establishing the trust in it.

Numerous publications have explored the application of AI in cancer research, precision medicine (23), cancer immunotherapy (24), and neoantigen identification (25). To gauge the potential of AI-driven software solutions, several benchmarking studies have been conducted. Evaluating and comparing tools is an essential part for their future application in the medical field and everyday clinical practice, as no single approach is universally applicable and having a dependable predictor or genotyper is vital. Despite the continually improving performance, critical questions regarding the application of AI technology in cancer immunotherapy remain. In this review, we summarize the core neoantigen calling pipeline, the recent research progress, and discuss the potential of artificial intelligence-enabled neoantigen identification, along with its current limitations and challenges.

## 2 Computational hunting for neoantigens

The core computational pipeline established for the process of identification and selection of genomically encoded antigens that are of immunological significance includes the following steps (25):

Whole exome or genome sequencing (WES or WGS) data of tumor and matched normal DNA samples
Somatic mutation calling
Conversion of detected coding DNA somatic mutations to corresponding mutated peptide sequences
HLA-allele typing
Peptide prioritization, neoantigen calling
o Prediction of peptide-MHC binding affinity
o Prediction of T cell receptor (TCR) recognition, TCR binding affinity and T cell response
o Immunogenicity prediction
o Expression analysis of putative neoantigens, using e.g. RNA-seq data

**Figure 2:**
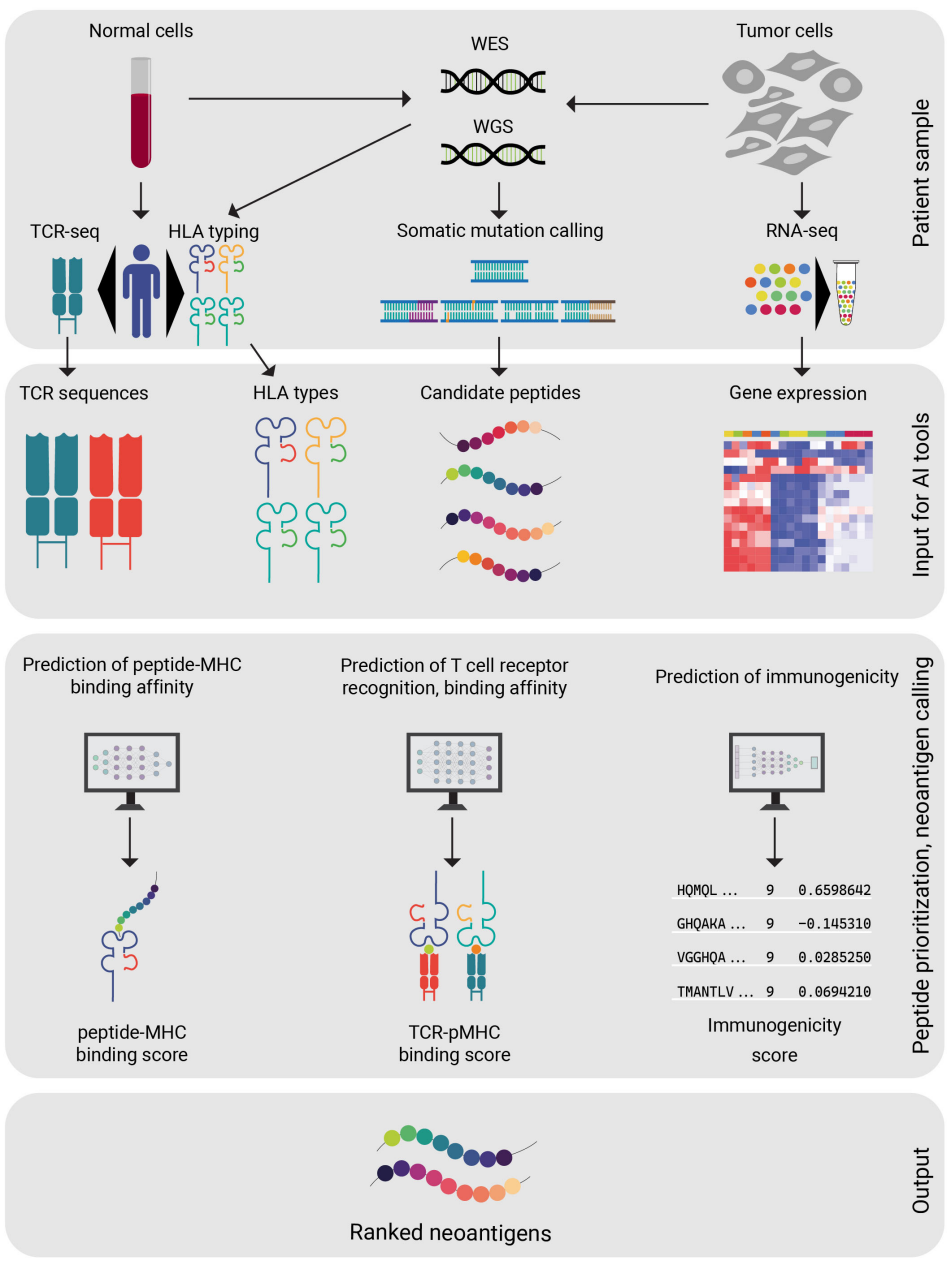
Steps of neoantigen selection from patient data. A set of diagnostic procedures are completed on patient derived samples. Ideally all of the above-mentioned patient data (WES, WGS, HLA typing, RNA-seq) are available before proceeding. After a candidate peptide selection is generated from the patient data, the AI model of preference is applied. The AI model will compute a ranked peptide list from the candidate peptides. Careful design of personalized vaccine is available, based on the peptide rankings.

The effective pattern recognition by AI allows for the development of personalized cancer treatments by considering the unique genomic profile of each patient’s tumor. As standard practice, neoantigens are predicted from the mutated peptides by assessing their ability to trigger an immune response. The development of AI-based prediction tools allows immunologists to streamline the search for neoantigen candidates that require experimental validation (**Figure 2**).

In the following, we provide an overview of the most common computational methods used in the neoantigen identification pipeline and outline the challenges associated with the process.

### 2.1 Somatic mutation calling

The process of somatic mutation calling is well-established and includes several critical steps, such as quality control of sequencing reads, alignment to the reference genome, base quality recalibration and INDEL realignment, comparison of healthy and tumor alignments. For quality control of sequencing reads in a WES (or WGS) dataset, FastQC (26) is commonly used, and BWA (27) is a widely employed aligner. Base quality recalibration and INDEL realignment around clusters of putative somatic mutations are both integral tools of Genome Analysis Toolkit (GATK) (28). There are numerous somatic mutation callers available, including MuTect (29), Abra (30), Strelka (31), and VarScan (32). For best practices in variant calling in clinical sequencing, readers are referred to the work of Koboldt (33). A comprehensive overview of the variant calling tools and their pros and cons is provided in the paper of Cai et al. (25).

Various databases can be used for variant annotation, such as CancerHotspots (34), and the Catalogue Of Somatic Mutations In Cancer COSMIC (35). The Variant Interpretation for Cancer Consortium (VICC) has standardized the curation, representation, and interpretation of clinically-relevant evidence associated with genomic variation in cancers. VICC guidelines (36) can be used to classify variants in known cancer genes (37).

### 2.2 False-positive mutation calls

There is a possibility that an identified mutation may yield a false-positive result potentially leading to the treatment of a patient with a drug targeting a nonexistent somatic mutation. To mitigate clinical efficacy risk, mutation calls from DNA sequencing should be cross-verified with the results of replicate sequencing runs. Moreover, utilizing extra sequencing data, like RNA-seq from the same tumor sample, to identify somatic mutations and check for overlaps reduces false positives. Yet, it may raise the risk of false negatives due to transient gene expression and variable read coverage (38). Combining multiple somatic mutation callers has been observed to significantly reduce the false positive rate (39, 40).

### 2.3 Identified mutation is a SNP

There is a possibility that an identified mutation exists in both tumor and healthy (germline) cells, representing a single nucleotide polymorphism (SNP) rather than a somatic mutation. Deep sequencing of germline DNA samples is essential to identify potential SNPs with high sensitivity.

### 2.4 False-negative mutation calls

There is a possibility that variant calling may fail to detect a somatic mutation that could produce a highly immunogenic neoantigen. While this omission does not harm the patient directly, it can result in a missed candidate neoantigen for the vaccine. To minimize this risk, deep sequencing of DNA samples (typically ~200x) is recommended to ensure high coverage across the entire protein-coding region. Unlike germline testing, which typically requires a minimum of 30x coverage with balanced reads, the identification of somatic variants in tumor specimens demands significantly higher read depths. This necessity arises from the presence of tissue heterogeneity, encompassing malignant cells, supporting stromal cells, inflammatory cells, and contaminant tissue. Additionally, intra-tumoral heterogeneity, represented by various tumor subclones, and considerations of tumor viability further underscore the need for elevated coverage. In instances of low tumor cellularity in tissue specimens, achieving an average coverage of at least 1000x may be essential to confidently detect heterogeneous variants. Additionally, the option to include multiple targets (e.g., up to 20 candidate neoantigens) in an individual drug product should limit the impact of missed mutations.

### 2.5 Sources of cancer neoantigens beyond single-nucleotide variants

Emerging evidence suggests the existence of alternative sources of cancer neoantigens, such as alternative splicing variants (41), post-translational modifications (42), and transposable elements (1), and gene fusions (43). These alternative sources may serve as attractive novel targets for immunotherapy (44). Nevertheless, addressing the tumor-specificity still remains a challenge.

## 3 HLA-allele typing

HLA typing of the individual patient samples, specifically the accurate identification of the individual set of HLA alleles (HLA allotypes), is essential. Peptide-MHC affinity strongly depends on HLA alleles, resulting in distinct immune responses among individuals (45). Genotyping the class I genes HLA-A, -B and -C, as well as the class II genes HLA-DRB1, -DQB1, and -DPB1 presents a non-trivial task.

Sequence-based typing (SBT) based on Sanger sequencing can be used for HLA typing. However, due to certain limitations, such as the need for additional sequencing to identify cis/trans polymorphism, the concordance rate of Sanger sequencing-based HLA genotyping is approximately 84% among different laboratories (46). Commercial software, such as uTYPE (Life Technologies. Brown Deer, WI), Assign-SBT (Conexio, San Francisco, CA) (47), and SBTEngine (GenDx, Utrecht, Netherlands) (48), along with some open-source tools, e.g. SOAPTyping (49) are capable of producing predictions from Sanger sequencing data. However, they are increasingly being replaced by NGS-based methods. High-throughput WES and RNA-seq sequencing data also serve as a foundation for HLA typing. Most HLA genotyping tools take NGS sequencing data as the input and output HLA types. The algorithms employed by the tools primarily differ in how they map sequencing reads to a panel of reference HLA allele sequences and the strategy they use to subsequently score candidate alleles (50).

**Figure 3:**
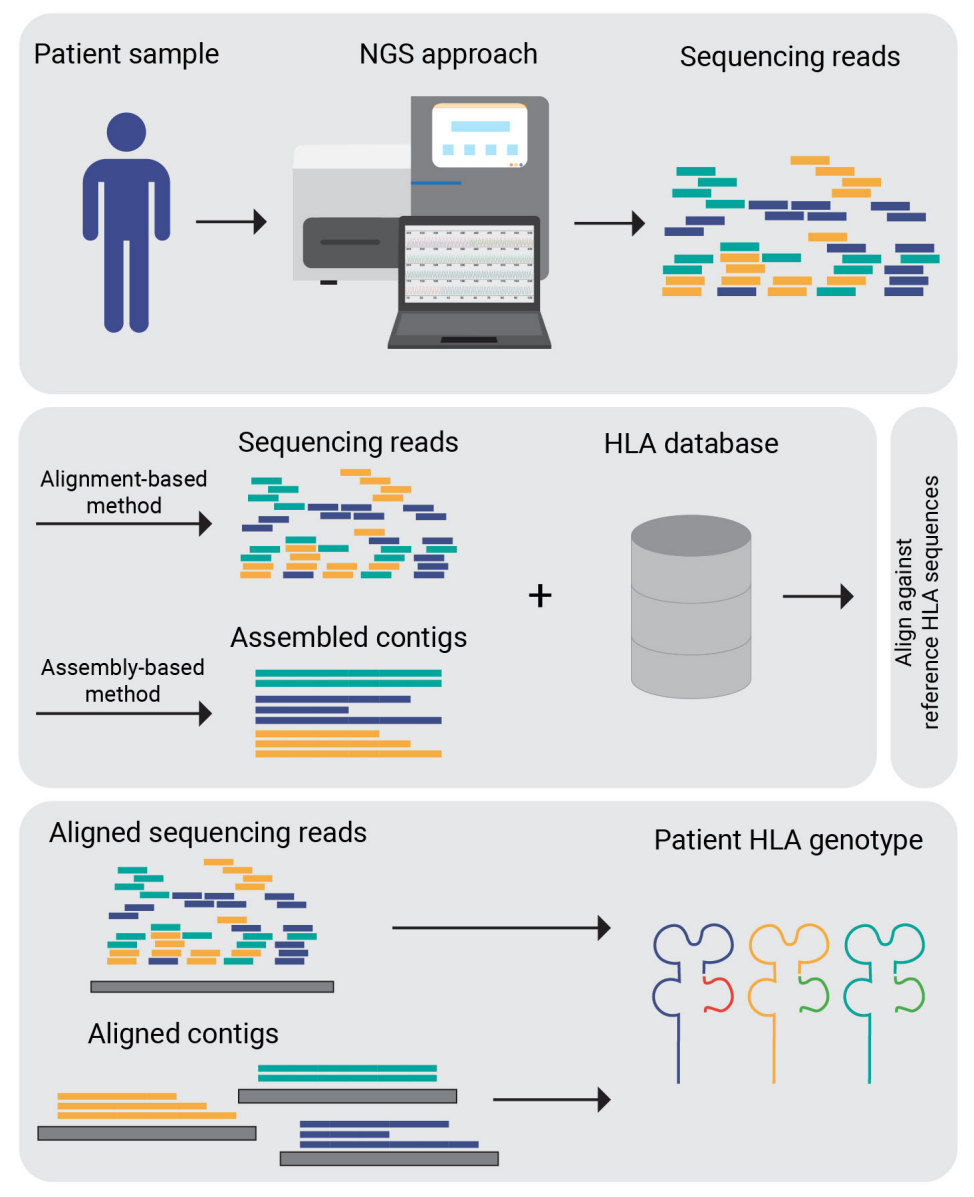
NGS-based HLA genotyping. Sequence data generated by sequencing technologies is mapped against the reference allele repository (IPD-IMGT). Corresponding to the HLA genotyping algorithm used either the raw reads or assembled contigs are aligned.

OptiType (51) is a HLA genotyping algorithm based on integer linear programming, capable of producing accurate 4-digit HLA genotyping predictions (for example, A01:01) from NGS data. To maximize the number of explained reads by simultaneously considering all major and minor HLA-I loci when predicting 4-digit HLA genotypes, this process involves aligning sequences from whole exome/genome/transcriptome sequencing data with a known MHC class I allele reference. Many tools for HLA typing are freely available for academic use, such as seq2HLA, ATHLATES, HLAminer, SOAP-HLA-2.2. A comprehensive list is provided in **Table 2**. **Figure 3** depicts a generalised workflow for NGS-based HLA genotyping.

**Table 2:** – HLA-allele typing.

HLA-allele typing			
Algorithm	Year	Input	URL
**seq2HLA**	2012 (52)	RNA-seq	https://github.com/TRON-Bioinformatics/seq2HLA
**HLAminer**	2012 (53)	WES/WGS/RNA-seq/Long Reads	http://www.bcgsc.ca/platform/bioinfo/software/hlaminer
**ATHLATES**	2013 (54)	WES	https://github.com/cliu32/athlates
**SOAP-HLA**	2013 (55)	Target capture sequencing/WGS	http://soap.genomics.org.cn/SOAP-HLA.html
**HLAforest**	2014 (56)	RNA-seq	https://code.google.com/p/hlaforest/
**OptiType**	2014 (51)	WES/WGS/RNA-seq	https://github.com/FRED-2/OptiType
**PHLAT**	2014 (57)	WES/WGS/RNA-seq	https://sites.google.com/site/phlatfortype
**hla-genotyper**	2014 (58)	WES/WGS/RNA-seq	https://pypi.org/project/hla-genotyper/
**HLAreporter**	2015 (59)	WES	http://paed.hku.hk/genome/
**POLYSOLVER**	2015 (60)	WES	http://www.broadinstitute.org/cancer/cga/polysolver
**HLA-VBSeq**	2015 (61)	WGS/WES	http://nagasakilab.csml.org/hla
**HLA-HD**	2017 (62)	WES/WGS/RNA-seq/Long reads	https://www.genome.med.kyoto-u.ac.jp/HLA-HD/
**xHLA**	2017 (63)	WGS/WES	https://github.com/humanlongevity/HLA
**Kourami**	2018 (64)	WGS/WES	https://github.com/Kingsford-Group/kourami
HLA*LA (HLA*PRG)	2019 (65)	WGS/WES	https://genomeinformatics.github.io/HLA-PRG-LA/
**ArcasHLA**	2020 (66)	RNA-seq	https://github.com/RabadanLab/arcasHLA

### 3.1 Benchmarking of HLA genotyping tools

There are multiple studies benchmarking HLA genotyping tools. Matey-Hernandez et al. (67) found that HLA typing tools based on WES and RNA-seq data exhibit prediction power almost equivalent to gold standards like PCR. Li X. et al. (45) focused on TCGA (68) cohorts, revealing superior performance of HLA class I over class II, with POLYSOLVER (60), OptiType (51) and xHLA (63) demonstrating high accuracy in HLA class I calling, and an ensemble HLA calling from the top-3 tools outperformed individual ones. Claeys et al.’s (69) study assessed 13 MHC class I and/or class II HLA callers, highlighting OptiType and arcasHLA (66) for MHC-I calling accuracy and HLA-HD (62) for MHC-II calling accuracy. The study concludes that the optimal HLA genotyping strategy from NGS data depends on factors like data type, dataset size, and computational resources, recommending OptiType and HLA-HD if resources permit (69).

## 4 Peptide-MHC binding prediction

T cells recognize peptides presented on MHC molecules of tumor cell. These molecules come in two main classes: peptide-MHC class I complexes, found on nucleated cells and recognized by CD8 + T cells, and peptide-MHC class II complexes, displayed on antigen-presenting cells like dendritic cells, activating CD4 + T cells. The diverse peptide repertoire is influenced by allele-specific amino acid preferences of MHC molecules. Due to individual variations in MHC alleles, the presented repertoire varies across people, with certain alleles being more common. The peptide-MHC interaction determines neoepitope presentation, impacting the level and type of T cell responses generated. While experimental MHC binding assays involve synthesizing and testing peptides, this is laborious and expensive on a large scale. Consequently, various computational algorithms and tools have been developed to predict peptide-MHC binding or assess binding affinity between mutated peptides and the patient’s MHC alleles (70).

It is important to note that other biologic processes can impact antigen presentation and immunogenicity of a particular neoantigen beyond MHC binding. Other factors, such as delivery of antigen to antigen presenting cells, antigen cleavage and processing by immunoproteasomes, peptide-MHC complex stability, are also important determinants of immunogenicity (7).

Early prediction tools relying on techniques as position-specific scoring matrices (PSSM) or sequence-scoring functions, such as SYFPEITHI (71), RANKPEP (72), PickPocket-1.1 (73), MixMHCpred (74), encountered difficulties in recognizing correlated effects. These effects manifest when an amino acid’s binding is influenced by the other amino acids in the peptide. The limitations of earlier tools in recognizing such correlated effects emphasize the suitability of neural networks as methods adept at considering these complex interactions (75).

Over the last decade, MS-based MHC peptidomics has become the dominant source of information about MHC binding specificities, with the ability to analyze ligands at greater depths than *in vitro* binding assays. Compilation of MHC ligandome data – the entirety of HLA presented peptides has been advanced by mass spectrometry (MS) based immunopeptidomics, in which the whole immunopeptidome of the cell is harvested and then eluted ligands (EL) are identified using MS. First application of direct neoepitope candidate identification using MS in native human tumors was presented in the paper of Bassani-Sternberg et al. (76). The authors assembled the ligandomes from human melanomas to a depth of 95,500 ligands. Eleven ligands were derived from candidate neoantigens, and four were proven to be immunogenic in T cell validation assays. MS profiling of HLA-associated peptidomes in mono-allelic cells enabled more accurate MHC-I epitope prediction in the study of Abelin et al. (77). MS immunopeptidomics is also able to identify protein hotspots, or regions within a protein prone to proteasomal cleavage and ligand production (78). Freudenmann et al. (79) constructed their own dataset and identified thousands of peptides bound to 16 different HLA class-I alleles to assess critical factors needed to epitope presentation.

However, in EL MS workflows typically pan- or locus-specific antibodies are used for immunoprecipitation (IP) during the purification of peptide–MHC complexes. This results in inherently poly-specific or Multi Allelic (MA) data, which comprises peptides that align with multiple cognate MHC binding motifs (80). For example, any of the six different MHC-I proteins present in a cell might be responsible for a peptide observation. These data need to be deconvoluted, i.e. transformed to Single Allelic (SA) or single peptide-MHC annotations, to be employed for the training of MHC-specific binding predictors. The method NNAlign_MA (81) resolved this limitation by incorporating into the prediction algorithm training procedure a strategy called *pseudolabeling*, which clustered EL sequences with ambiguous cognate MHCs into single MHC specificities.

Various AI-based tools have been developed to predict peptide-MHC binding using a range of neural network architectures and strategies in an attempt to improve predictive performance and generalizability of their models. They work on multiple data types including peptide sequences and mass spectrometry profiles.

One major issue impeding the generalizability of ML models is the lack of binding affinity data for rare MHC alleles. This can be addressed using various approaches such as using the sequence homology of rare MHC alleles with common MHC alleles to infer potential ligand preferences as NetMHCpan (82, 83) does. Also, NNAlign_MA was deployed in NetMHCpan to deconvolute ligandomes from MS datasets (80).

Another way is to use transfer learning by pre-training models on more common MHC classes and fine-tuning the models on the data for rare MHC classes. This approach is used by tools such as MHCnuggets (84), ImmunoBERT (85) and MHCRoBERTa (22). ImmunoBERT used transfer learning from the Tasks Assessing Protein Embeddings (TAPE) (86). The TAPE model was trained on a dataset of over 31 million protein sequences from the Pfam database. The authors of MHCRoBERTa used self-supervised training with label-agnostic protein sequences from UniProtKB (87) and Swiss-prot databases, and then fine-tuned the training with data from the Immune Epitope Database and Analysis Resource (IEDB) (88).

Many tools use approaches adopted from other domains. From the image processing domain comes the convolutional neural network which can learn multiple intrinsic features of the peptide sequence that can be used to predict binding affinity. Examples of these tools include ConvMHC (89), HLA-CNN (90) and DeepMHC (91). MHCSeqNet (92) uses techniques from the natural language processing domain by treating epitope peptide sequences as sentences composed from amino acids as individual words.

Some tools use ensemble learning, a technique that combines the output of several models using a weighted or uniform consensus. The concept behind the consensus methods is that prediction performance can be further improved by integrating the outputs from several individual tools using a weighted scheme. This includes tools such as MHCflurry (93) and NetMHCcons (94). MHCflurry is supporting only a fixed set of alleles (95).

Others tools provide or require additional data. Tools such as HABIT (96) provides an interpretation of the impact of amino acid variants alongside the binding affinity prediction. EDGE (97) and MARIA (98) require transcript abundances and flanking sequence in addition to the peptide sequence and MHC allele.

A class of tool use mass spectrometry and immunopeptidomics data as input data instead of peptide sequence data. This class of tool includes HLAthena (99) which shows 1.5-fold enhanced accuracy compared to sequence based tools and SHERPA (100).

An overview of tools used for MHC binding prediction is shown in **Table 3**.

**Table 3:** – Peptide-MHC binding affinity prediction.

Peptide-MHC binding affinity prediction				
Algorithm	Year	Strategy	MHC	URL
**NetMHC-4.0**	2016 (101)	Gapped sequence alignment using ANN	MHC-I	https://services.healthtech.dtu.dk/services/NetMHC-4.0/
**MixMHCpred 1.0**	2017 (74)	Fully unsupervised and semi-supervised ML	MHC-I	Only updated version is available at: https://github.com/GfellerLab/MixMHCpred
**ConvMHC**	2017 (89)	DCNN	MHC-I	https://github.com/aidanbio/convmhc
**HLA-CNN**	2017 (90)	DCNN	MHC-I	https://github.com/uci-cbcl/HLA-bind
**NetMHCpan-4.0**	2017 (83)	ANN	MHC-I	https://services.healthtech.dtu.dk/services/NetMHCpan-4.0/
**DeepMHC**	2017 (91)	DCNN	MHC-I	http://mleg.cse.sc.edu/deepMHC/
**MHCflurry**	2018 (93)	ANN	MHC-I	Only updated version is available at: https://github.com/openvax/mhcflurry
**AI-MHC**	2018 (102)	DCNN	MHC-I MHC-II	https://baras.pathology.jhu.edu/AI-MHC/index.html
**MHCSeqNet**	2019 (92)	DCNN	MHC-I	https://github.com/cmbcu/MHCSeqNet
**EDGE**	2019 (97)	DCNN	MHC-I	Not available
**MARIA**	2019 (98)	RNN	MHC-II	https://maria.stanford.edu/
**DeepHLApan**	2019 (103)	GRU combined with attention	MHC-I	http://biopharm.zju.edu.cn/deephlapan
**CNN-NF**	2019 (104)	DCNN	MHC-I	https://github.com/zty2009/MHC-I
**DeepLigand**	2019 (15)	Deep language model (ELMo) pre-trained on natural ligands, combined with deep residual network	MHC-I	https://github.com/gifford-lab/DeepLigand
**PUFFIN**	2019 (105)	Deep residual network-based approach that quantifies uncertainty in prediction	MHC-I MHC-II	https://github.com/gifford-lab/PUFFIN
**NeonMHC2**	2019 (106)	Ensemble of CNNs	MHC-II	https://neonmhc2.org/
**MHCherryPan**	2019 (107)	LSTM, CNN	MHC-I	Not available
**DeepSeqPan**	2019 (108)	DCNN	MHC-I	https://github.com/pcpLiu/DeepSeqPan
**DeepSeqPanII**	2019 (109)	RNN combined with attention	MHC-II	https://github.com/pcpLiu/DeepSeqPanII
**ACME**	2019 (110)	Attention-based CNNs	MHC-I	https://github.com/HYsxe/ACME
**MHCnuggets**	2020 (84)	LSTM networks and GRUs	MHC-I MHC-II	https://github.com/KarchinLab/mhcnuggets
**USMPep**	2020 (111)	Learned embedding layer; AWD LSTM with one hidden layer	MHC-I MHC-II	https://github.com/nstrodt/USMPep
**IConMHC**	2020 (112)	DCNN	MHC-I	Not available
**MHCAttnNet**	2020 (113)	Attention-based deep neural model, MHC alleles classes I and II	MHC-I MHC-II	https://github.com/gopuvenkat/MHCAttnNet
**MHCflurry 2.0**	2020 (95)	ANN	MHC-I	https://github.com/openvax/mhcflurry
**NetMHCpan 4.1**	2020 (80)	ANN	MHC-I	https://services.healthtech.dtu.dk/services/NetMHCpan-4.1/
**BERTMHC**	2021 (21)	BERT-based architecture and multiple instance learning	MHC-II	https://bertmhc.privacy.nlehd.de/, https://github.com/s6juncheng/BERTMHC
**DeepAttentionPan**	2021 (114)	DL pan-specific model with improved attention mechanism	MHC-I	https://github.com/jjin49/DeepAttentionPan
**DeepNetBim**	2021 (115)	DL model based on network analysis by harnessing binding and immunogenicity information	MHC-I	https://github.com/Li-Lab-SJTU/DeepNetBim
**SHERPA**	2021 (100)	Composite model incorporating binding affinity, monoallelic and multiallelic data constructed with gradient boosting decision trees	MHC-I	Not available
**MATHLA**	2021 (116)	Bidirectional LSTM and multiple head attention mechanism	MHC-I	https://github.com/MATHLAtools/
**ImmunoBERT**	2021 (85)	BERT-based architecture	MHC-I	https://github.com/hcgasser/ImmunoBERT
**MHCRoBERTa**	2022 (22)	Pan-specific prediction through transfer learning with label-agnostic protein sequences	MHC-I	https://github.com/FuxuWang/MHCRoBERTa
**FIONA**	2022 (117)	Flexible Immunogenicity Optimization NN Architecture	MHC-II	http://therarna.cn/fiona.html
**HLApollo**	2022 (118)	Transformer model with diverse negative coverage, deconvolution and protein language features	MHC-I	Not available
**HLAB**	2022 (119)	BiLSTM feature learning from ProtBert-encoded proteins	MHC-I	http://www.healthinformaticslab.org/supp/resources.php
**DeepNeo**	2023 (120)	DCNN	MHC-I MHC-II	https://deepneo.net/
**IEPAPI**	2023 (121)	Transformer-based feature extraction, incorporating antigen presentation and immunogenicity	MHC-I	https://github.com/ddd9898/IEPAPI
**MixMHC2pred 2.0**	2023 (122)	Deep motif deconvolution with MoDec, fully connected NNs	MHC-II	http://mixmhc2pred.gfellerlab.org/
**CapsNet-MHC**	2023 (123)	Capsule neural networks	MHC-I	https://github.com/s7776d/CapsNet-MHC
**DeepMHCI**	2023 (124)	Anchor position-aware deep interaction model	MHC-I	https://github.com/ZhuLab-Fudan/DeepMHCI
**MixMHCpred 2.2**	2023 (125)	Fully unsupervised and semi-supervised ML	MHC-I	https://github.com/GfellerLab/MixMHCpred
**TLimmuno2**	2023 (126)	MHC class II antigen immunogenicity through transfer learning	MHC-II	https://github.com/XSLiuLab/TLimmuno2
**NetMHCIIpan-4.2**	2023 (127)	ANN	MHC-II	https://services.healthtech.dtu.dk/services/NetMHCIIpan-4.2/

Other tools focus on visualizing and comparing different MHC molecule binding specificities to aid the understanding of main binding properties An example of such as tool is MHC Motif Atlas (128, 129) which contains 1,013,733 ligands interacting with 135 MHC-I and 88 MHC-II molecules, including information about binding motifs, peptide length distributions, motifs of phosphorylated ligands, multiple specificities and enables users to download curated datasets of MHC ligands, MHC sequences and MHC X-ray crystallography structures.

### 4.1 Identification of MHC class II neoantigens is challenging

Predicting MHC class II binding poses an extra challenge compared to class I due to limited training data and the complex nature of HLA-II ligands. In humans, HLA class II is encoded by three different loci (HLA-DR, -DQ, and -DP) with numerous allelic variants and polymorphisms clustered around the peptide-binding groove, resulting in a wide range of distinct peptide binding specificities. This complexity of HLA-II ligands results in binders with longer and more heterogeneous peptide sequences and varying peptide length distributions, making their prediction more challenging (106, 130). A comprehensive trans-allelic model for prediction of peptide-MHC-II interactions for all three human MHC-II loci was proposed by Degoot et al. (131). The authors investigated contributions of certain binding pockets to the binding energy and found that binding pocket P5 of HLA-DP contributes strongly to the binding energy. Most HLA class II prediction algorithms have primarily targeted HLA-DR molecules, given the extensive data available for them (127). On the other hand, HLA-DQ molecules are more complex to study experimentally.

NetMHCIIpan-3.2 (132) and NetMHCIIpan-4.0 (80) predict antigen presentation for any HLA class II molecule. For HLA-DQ and DP heterodimers, both α- and β-chain sequences are needed. Nilsson et al. (127) used a DQ-specific antibody during purification to obtain immunopeptidome data for 14 different HLA-DQ molecules from 16 homozygous B Lymphoblastoid Cell Lines (BLCLs) using liquid chromatography coupled with mass spectrometry (LC-MS/MS) to train NetMHCIIpan-4.2. Benchmarked against MixMHC2pred-2.0 (122), on independent DQ data consisting of EL data from 15 donor samples enriched with random negative peptides, NetMHCIIpan-4.2 excelled in motif deconvolution and identifying DQ ligands. BERTMHC is an transformer-based peptide-MHC class II interaction prediction method (21). The pretrained BERT from TAPE repository was used to model the input amino acid sequences. Additionally, multiple instance learning was employed to account for the limitation that mass spectrometry data often cannot precisely identify the exact MHC molecule to which a peptide was bound.

Four methods (MHCnuggets (133), AI-MHC (102), PUFFIN (105), and USMPep (111)) can make predictions for both MHC classes. A majority of the responses to neoantigens in preclinical and clinical setting are MHC class II restricted (134). Therefore, improvement of algorithms on MHC class II binding interactions is crucial, since it will significantly enhance the selection of MHC-class II restricted neoantigens.

### 4.2 Challenges of mass spectrometry limiting MHC ligandome datasets

MS data has inherent biases such as overrepresentation of “flyable” peptides and neglect of cysteine-containing peptides, limiting the detectable set of ligands (80). Some MHC molecules, such as HLA-C and HLA-DQ, have limited ligand datasets (80). The performance of AI-based approaches used for predictions relies on quality and diversity of the training data. Therefore,high-quality data sets covering a broad range of HLA alleles, are crucial. Future work exploiting antibodies with improved specificities or using engineered cell lines with tagged HLA molecules might help to resolve this.

### 4.3 Benchmarking of peptide-MHC binding prediction tools

Benchmarking peptide-MHC binding predictors is not straightforward due to differences in the MHC alleles, peptide sizes, and non-standardized outputs of the methods. In 2014, the Immune Epitope Database automated benchmark was established to address the need for an unbiased evaluation of the MHC-I binding predictors (135). They assembled a blind test which ensures that the data will be new to all of the participating tools (135, 136). Based on the criteria established by the benchmark a peptide is deemed a binder if it was experimentally reported to qualitatively bind to an MHC, or its half-life (T1/2) bound to the MHC is reported to be longer than 120 min, or its IC50 is reported to be lower than 500 nM (135). Peptides that do not meet any of those criteria are considered non-binders (137).

Trevizani et al. (137) investigated predictor rankings using a benchmark. They found that due to the benchmark’s data update rate, a new method had to wait at least four years to be compared with existing ones. The top-performing tools consist of NetMHCcons-1.1, NetMHCpan-4.0, ANN 3.4 (138) (updated to ANN 4.0 (101) in 2016), NetMHCpan-3.0 (82) and NetMHCpan-2.8 (139), with statistically indistinguishable scores. The authors also determined that using percentile-ranked results from original metrics provided reliable rankings across different data sets.

Another comprehensive performance assessment of 15 *in silico* tools for MHC class I peptide binding prediction, including 6 scoring function-based, 7 ML-based and 2 consensus methods, was described in Mei et al. (140). Extensive benchmarking tests showed that MixMHCpred (141) performs best across most HLA-I allotypes, while NetMHCpan and NetMHCcons achieve the overall best performance among ML-based and consensus-based tools.

## 5 T cell receptor recognition

T cell receptors (TCRs) play a pivotal role in surveillance and response to disease by recognizing peptide-MHC (pMHC) complexes. However, not all neoantigen candidates elicit an immune response from T cells even though they are expressed and presented on the cell surface (11). Understanding the rules governing how T cells recognize cognate antigen-MHC complexes remains a challenge in systems immunology.

The TCR is a heterodimeric protein comprising an α- and β-chain. Peptide specificity is primarily defined by the complementarity-determining region 3 (CDR3) loops. The diversity of the CDR3s results from genomic recombination of the variable (V), diversity (D), and joining (J) genes (142). The majority of previous studies have focused on the β-chain alone due to its higher diversity, resulting from the V-, D-, J genes together (142). In contrast, the α-chain results from V- and J recombination which leads to lower diversity and less interest. However recent research has highlighted the importance of both α- and β-chain CDR3s in TCR specificity (143, 144).

T cell receptor sequencing (TCR-Seq) is an NGS approach allowing scientists to study clonal expansion by selectively amplifying and sequencing antigen-specific CDR3 regions of the T cell receptor. However, TCR-Seq data analytics is challenging as tumor-specific T cell responses constitute a small proportion of the overall pool of *in vivo* T cell responses with irrelevant specificities (145). New analytical tools have been developed to parse and draw meaningful sequence concepts or motifs from the TCR-Seq data (146). The TCRdb database contains more than 277 million TCR sequences from over 8265 TCR-Seq samples across hundreds of tissues, clinical conditions and cell types (147).

Assessing the interactions between neoepitopes and TCRs is essential for designing immunotherapies. For instance, identifying compatible TCRs in the patient’s circulation can help inform the selection of neoantigen vaccine candidates. Various experimental approaches, such as tetramer analysis (148), TetTCR-seq (149) and T-scan (150), have been developed to detect pairing of TCR–pMHC complexes. However, *in vitro* experiments associated with the testing of a large number of putative candidates demand experimental time and costs.

TCRdist (143) represents an unsupervised distance-based method exploiting the similarity between TCRs to produce clusters of TCR sequences that likely recognize the same antigen, and predicting binding for a given epitope sequence. The methods like TCRex (151) and DeepTCR (152) trained antigen-specific TCR models and would have problems to generalize to unseen peptides. In response, the scientific community has turned to ML and AI-based approaches to develop computational solutions for TCRs and peptide–MHC and TCR–peptide interaction prediction.

NetTCR (153) facilitates sequence-based prediction of TCR binding to pMHC complexes using CNNs. CNN is an appropriate model to handle unaligned peptide and TCR sequences differing in length. The model was trained on the IEDB data, containing TCR β-chain CDR3 sequences and corresponding peptide targets presented by most common MHC-I HLA-A*02:01 allele. Negative data examples were supplied for the learning by generating wrong combinations of TCRs and peptides, and additional negatives constructed from the TCRs of healthy donors. For NetTCR-2.0 (142) is a “shallow” CNN model, similar to NetTCR (153), it was exploited, but trained on paired TCR α and β chain sequence data. Nonbinding peptide-CDR3β pairs derived from 10X Genomics (154) Chromium Single Cell Immune Profiling of four donors were used as negative data set. The model has the potential to infer not only which TCRs are specific for a given peptide, but also which peptide is specific for a given TCR. This work also underlined the need for technologies for high-throughput paired sequencing of TCRs with known pMHC targets. The current optimal way to pair TCR α- and β- chain is through single-cell TCR sequencing (scTCR-Seq) (155). The authors of NetTCR-2.1 (156) provide lessons and guidance on how to develop models for TCR specificity predictions, how to best define negative data, and why it is recommended to apply similarity-based modeling, and include a performance evaluation as a function of “distance” to the training data when validating predictive power of ML-based approaches.

Most of the peptides in the published databases originate from viruses but not from tumor-associated antigens and there are only a few CDR3α sequences in databases available. Therefore, AI-driven approaches with improved generalization ability are needed, which do not show significant performance drop when evaluated on peptide sequences not used during model training. This challenge can be addressed by approaches based on *transfer learning* and NLP, capable to benefit from unsupervised pre-training.

As an example for the application of a newly emerging DL approach, Lu et al. (157) used *transfer learning* to develop pMTnet, a model predicting the TCR binding specificity of class I pMHCs. Utilizing the “Atchley factor” (158) they encoded TCR CDR3β sequences with five numeric values per amino acid, providing comprehensive biochemical characterization. These “Atchley matrices” were input into a stacked auto-encoder, an effective unsupervised learning algorithm. During training, the auto-encoder reconstructed input data, generating a 30-neuron numeric vector that encapsulates the inherent structure of the original CDR3s. The embedding of pMHCs closely followed the NetMHCpan algorithm. Fixed numeric encodings of TCRs and pMHCs were integrated into a DL network with a single neuron as the final layer for pairing prediction. To train this model, Lu et al. (157) employed a differential learning schema, using known interactions as positive data and introducing true and mismatched pairs for negative data, resulting in ten times more negative data by randomly mismatching TCRs and pMHCs. This approach allowed them to capitalize on a substantial volume of related TCR and pMHC data without explicit pairing information, showcasing the effectiveness of transfer learning.

For their NLP-based approach BERTrand (159) the authors constructed a hypothetical human TCR-peptide repertoire pre-training set comprising peptides from MHC-I MS peptide presentation experiments and TCRs from healthy donors, and this hypothetical TCR-peptide repertoire was used to perform masked language modeling (MLM), pre-training of the BERT model. Then the pre-trained BERT model was fine-tuned to predict TCR-peptide binding using the dataset of known TCR binders with their cognate epitopes and negative decoy examples generated by random pairing of reference TCRs with peptides. ERGO (pEptide tcR matchinG predictiOn) (160) and ERGO-II (161) utilize unsupervised TCR pre-training and use a pre-trained LSTM neural network architecture.

Further published tools for TCR-pMHC binding prediction are shown in our **Table 4**.

**Table 4:** – TCR-pMHC binding prediction.

TCR-pMHC binding prediction			
Algorithm	Year	Strategy	URL
**TCRdist**	2017 (143)	Distance-based clustering of similar TCRs	https://github.com/phbradley/tcr-dist
**TCRex**	2019 (151)	Random Forest algorithm based on epitope-specific TCR data	https://tcrex.biodatamining.be
**ERGO-I**	2020 (160)	Embeds TCR and peptide by LSTM and autoencoder followed by fully connected NNs for pattern learning	https://github.com/louzounlab/ERGO
**ERGO-II**	2021 (161)	Extends embedding of ERGO-I	https://github.com/louzounlab/ERGO
**DLpTCR**	2021 (162)	Ensemble DL framework from FCN, CNN and ResNet	http://jianglab.org.cn/DLpTCR/
**NetTCR-2.0**	2021 (142)	DCNN	https://services.healthtech.dtu.dk/service.php?NetTCR-2.1
**TCRAI**	2021 (163)	Binary classification including embedding layers and convolutional networks to predict TCR-pMHC–specific binding	https://github.com/regeneron-mpds/TCRAI
**TCRGP**	2021 (164)	Gaussian process classification, utilize CDR sequences from both TCRα and TCRβ chains, single-cell RNA-sequencing analysis of HCC-patients	https://github.com/emmijokinen/TCRGP
**pMTnet**	2021 (157)	LSTM and autoencoder followed by fully connected NNs	https://github.com/tianshilu/pMTnet
**ImRex**	2021 (165)	DCNN using interaction maps representing TCR CDR3 and epitope sequences	https://github.com/pmoris/ImRex
**TITAN**	2021 (166)	Attention-based NNs pretrained with BindingDB	https://github.com/PaccMann/TITAN
**DeepTCR**	2021 (152)	DCNN	https://github.com/sidhomj/DeepTCR
**AttnTAP**	2022 (167)	Attention-based dual-input DL framework	https://github.com/Bioinformatics7181/AttnTAP/
**ATM-TCR**	2022 (168)	Attention-based NNs	https://github.com/Lee-CBG/ATM-TCR
**epiTCR**	2023 (169)	Random Forest	https://github.com/ddiem-ri-4D/epiTCR
**DeepMHCI**	2023 (124)	Anchor position-aware deep interaction model	https://github.com/ZhuLab-Fudan/DeepMHCI
**iTCep**	2023 (170)	DL framework using fusion features derived from a feature-level fusion strategy	http://biostatistics.online/iTCep/, https://github.com/kbvstmd/iTCep/
**BERTrand**	2023 (159)	BERT model augmented with hypothetical random TCR pairing	https://github.com/SFGLab/bertrand

### 5.1 Limitations of current data sets for TCR–peptide binding prediction

Current datasets for TCR-peptide binding prediction present challenges for the development of accurate and generalizable models. As discussed in the perspective article of Hudson et al. (171), the current data sets cover only a limited fraction of the universe of possible TCR–antigen binding pairs. These datasets also inadequately represent the universe of self and pathogenic epitopes and of the varied MHC contexts in which they may be presented. Furthermore, a significant proportion of known antigens reported as binding a TCR are of viral origin, limiting their relevance to human health.

Current sources of publicly available data for AI-based methods to predict the interaction between TCR and pMHC complexes include manually curated catalogs of pathology-associated TCR sequences such as McPAS-TCR (172), Immune Epitope Database IEDB (88), VDJdb (173), and TBAdb (174) databases. Additionally, positive data samples generated by Klinger et al. (175), known as the MIRA set, are publicly available in the NetTCR-2.0 repository (176). For successful training and development, balanced training data is required. However, the publicly available datasets of TCR-pMHC sequences almost exclusively contain examples of positive binding pairs. Only the published 10X Genomics dataset contains both positive and negative data points. The choice of negative data is a critical factor when developing a binary classification model. NetTCR and pMTnet chose 10X Genomics Immune Profiling data, which contains validated non-binding complexes. Swapped negatives are randomly generated negative data, generated by other prediction tools (TCRGP (164), ERGO-I, ERGO-II, TITAN (166)), by mispairing positive validated TCR–peptide pairs. However, this approach risks to introduce false non-bindings into the ground truth.

In the future, as high-throughput technologies such as T-scan and 10X Immune Profiling are becoming more prevalent, it is expected that more training data for TCR-pMHC pairing will be available, providing a more accurate representation of the entire space of potential epitopes for training. Frank et al. (177) provide an overview of TCR sequencing platforms and the T cell repertoire analysis methods.

### 5.2 TCR binding predictors fail to generalize to unseen peptides

While many TCR-pMHC binding prediction methods perform well with test sets containing peptides from the training set, the ability to generalize to unseen peptides is crucial for neoantigen-based cancer vaccine development. Grazioli et al. (178) investigated the impact of various training/test splitting techniques on models’ test performance. They introduced Tchard, a sample collection with positive samples from the databases IEDB, VDJdb, McPAS-TCR, and the MIRA, along with negative samples from randomization and 10X Genomics assays. After ensuring that testing samples were not present in the training dataset, they found that modern DL methods may struggle with generalization to unseen peptides. Deng et al. (179) addressed this by comparing the performance of different TCR-pMHC prediction tools on various datasets. Regardless of model complexity, all tools, including TITAN, NetTCR-2.0, ERGO, DLpTCR and ImRex, faced challenges predicting unseen peptide examples. These challenges emphasize the necessity for ongoing research to enhance the generalization of TCR-pMHC binding predictors across a wider range of peptides.

## 6 Criteria for epitope selection

Only a small fraction of predicted neoepitopes can be experimentally validated in vitro as true neoepitopes (180). Several general criteria are currently employed in the field to narrow down and prioritize the candidate epitopes. These criteria guide the selection of epitopes to induce specific “on target” immunogenic response while overcoming self-tolerance.

### 6.1 MHC binding affinity

Mutant peptides must be presented by MHC-I or MHC-II in order to be recognized by T cells. Most neoantigen prioritization pipelines typically use the output values of the MHC-I or MHC-II binding prediction methods as the primary ranking parameter. The generally used MHC binding affinity threshold type is IC50 (half maximum inhibition concentration) measured in nM. The lower value shows stronger binding affinity. Usual thresholds are IC50 ≤ 50nM (strong) and IC50 ≤ 500nM (low). Another threshold type is percentile rank (%-rank) which allows to better compare scores between MHC molecules. Usually %-rank ≤ 0.5 is strong affinity and %-rank ≤ 2 shows lower affinity. NetMHCpan-4.1 differentiates %-rank prediction based on either LC-MS eluted ligands (EL) or binding affinity (BA). The third type is Score, as in SYFPEITHI (71). They typically do not recommend any threshold. Here, the higher binding score shows increased chances of binding.

It is important to note that these commonly used threshold values for identifying potential binders can be excessively strict in many cases (76) that can result in missing potential binders. To improve the sensitivity and accuracy of 13 already existing prediction tools Bonsack et al. (181) calculated new thresholds, recommended for each of them. They also developed MHCcombine (182) to facilitate the application of their prediction-improving recommendations and also to simultaneously compare the outputs of the selected predictors.

### 6.2 TCR binding affinity

As mentioned before, the T cell recognition and activation is a vital part of the immune response. In order to trigger immune response T cells need to recognize the peptides presented by the MHC molecules. Addressing the T cell activation outcome still remains challenging however generally can be determined based on the biochemical parameters of the pMHC-TCR interaction (11). The mostly used parameter is TCR-pMHC binding affinity. Gálvez et al. (183) aimed to uncover the shaping forces behind the TCR binding affinity with 12 phenotypic models and as a result they provide valuable insight and observations in the field of TCR binding affinity. As described in the review by Schaap-Johansen et al. (11) a number of structure-based methods have been developed lately which can greatly improve the overall TCR binding predictions by reducing the false positive predictions.

### 6.3 Agretopicity

The *differential agretopicity index* (DAI) has been proposed as a neoantigen quality metric (184). DAI is a property of the epitope and defined as the numerical difference between the NetMHC (138) scores of the WT peptides and their mutated counterparts (184). In an study of 6,324 patients across 27 cancer types, Rech et al. (185) found that high DAI neoantigens correlated with patient survival. The work of Ghorani et al. (186) also supported the hypothesis that DAI is a determinant of cancer peptide immunogenicity, by investigating the association between mean DAI, survival, and measures of immune activity.

### 6.4 Binding stability

Assuming that a more stable epitope presentation on the MHC increases the likelihood of T cell recognition, peptide stability, measured as the half-life of the binding interaction in units of hours, has been postulated to correlate with immunogenicity. Tools such as NetMHCstabpan (187) are often used in epitope selection pipelines to assess binding stability. Borden et al. (188) used a model-based approach to find the neoantigen properties that have predictive value of immunogenicity. The binding stability of the pMHC class I complex, along with the dissociation constant and the expression (mRNA and variant allele frequency) were the characteristics that were of predictive value. These findings were in consistence with previous studies (189). The authors integrated binding stability together with other factors such as neoantigen expression level and dissociation constant into an immunogenicity score called NeoScore (188).

### 6.5 Differential expression between tumor and healthy tissue

In contrast to pathogens seen as foreign invaders, most epitopes presented on the cancer cell surface are self-peptides unrecognized by tumor immunosurveillance. Neoepitopes, typically absent in benign tissues, may escape tolerance and become immunogenic. Databases such as TissGDB (190), GTEx (191), TCGA (68), THPA (192, 193) can be consulted to compare gene expression between healthy and tumor tissues, identifying cancer-specific signatures (194).

### 6.6 Dissimilarity to the self-proteome

As observed in the literature, sequence dissimilarity to non-mutated proteome was predictive of peptide immunogenicity (195, 196). Devlin et al. (197) demonstrated that structural dissimilarity between the wildtype and mutated peptide in non-anchor positions can influence T cell recognition and immunogenicity.

### 6.7 Expression of a peptide source gene in thymocytes

Medullary thymic epithelial cells (mTEC) contribute to the development of T cell tolerance by facilitating the recognition of “self” and expressing tissue-restricted antigens (TRA) (198). This allows developing T cells to assess the self-reactivity of their antigen receptors before leaving the thymus (198). The expression of a peptide source gene in mTEC is considered as a negative characteristic for epitope selection, as it may decrease the chances of immunogenicity due to the central tolerance.

### 6.8 Hydrophobicity

As described in the methods of TESLA consortium, the number of hydrophobic residues in the neoantigen can be divided by the total number of residues to create a “hydrophobicity fraction” (189). Additionally, the grand average of hydropathicity index (GRAVY) is used to estimate the hydrophobicity of a given amino acid string and is calculated as the average of the hydrophobicity of the individual residues forming the peptide (199). Immunogenic pMHC were significantly less hydrophobic than non-immunogenic pMHC (199).

### 6.9 Clonality

Clonality refers to the fraction of the tumor containing the neoantigen of interest and of particular importance for prioritization. The presence of a variant expressed by a small, sub-clonal population of the tumor makes it less attractive candidate for tumor therapy (200). In the review of Lang et al. (201) the impact of clonality on neoantigen recognition is discussed. Depending on whether the neoantigen is truncal clonal, truncal clonal but lost in a metastasis (by deletion or gene silencing), clonal in a certain metastasis (or specific for a certain subclone within a single metastasis), neoepitope-specific T cells would target either all tumor cells, all tumor cells of selected lesions, or merely a single tumor subclone (201). The tools PyClone (202) and its improved version PyClone-VI (203) provide a numerical estimation of cancer cell fraction using observed alternate allele frequencies, copy number, and loss of heterozygosity (LOH) information.

Other characteristics associated with immune response, such as the variant allele frequency of mutations, the number of predicted neoepitopes per mutation, peptide proteasomal cleavage probability, potential for TAP transport in the endoplasmic reticulum, tumor heterogeneity and HLA loss of heterozygosity (LOH), are used to further rank candidate neoantigens (200).

## 7 Integrated software for neoantigen detection and prioritization

Several integrated software and comprehensive pipelines have been developed for tumor-specific neoantigen detection. The purpose of these tools is to make the prediction and prioritization of neoantigen candidates accessible. Here, we describe some of the notable tools and frameworks and their approaches.

For seamless vaccine design there have been several end-to-end pipelines developed. One of the frequently used end-to-end pipelines is FRED2 (FRamework for Epitope Detection), a Python-based immunoinformatic framework (204). Among the included tools there are several HLA genotyping tools (e.g.: OptiType), as well as peptide-MHC binding predictors (e.g.: NetMHCpan, NetMHCIIpan), and also the proteasomal cleavage predictor NetChop (205) is integrated. FRED2 ensures straightforward workflow and provides analysis tools to epitope detection and vaccine design (204). Another end-to-end pipeline is pVACtools, which produces an end-to-end solution for neoantigen characterization (206). To aid the vaccine design, pVACtools supports the identification of altered peptides and prioritizes them by incorporating various data sources, such as clonality of the mutation, mutant allele expression and peptide binding affinities. Among the tools integrated inside pVACtools there are binding predictors (e.g.: MHCflurry), databases (e.g.: IEDB), and a proteasomal cleavage predictor (NetChop). To extract neoepitopes from tumor sequencing data such as VCF files and expression files generated from RNA-seq, MuPeXI (Mutant peptide extractor and informer) provides a prioritization suggestion based on a combined score named priority score (207). It generates an output file with the list of mutated peptides and all the information needed (expression level, similarities to self-peptides, mutant allele frequency) to select the peptides for vaccine design (207). For HLA binding prediction NetMHCpan is integrated. It is a web-based tool, and also available as a command-line tool. TIminer is also a computational framework that provides complex immunogenomic analysis including HLA typing (Optitype), neoantigen prediction (NetMHCpan), characterization of immune infiltrates and quantification of tumor immunogenicity (208).

Another solution for peptide design includes prioritization algorithms. One such predictor is PRIME (predictor of immunogenic epitopes) (209). It captures molecular properties of both antigen presentation and TCR recognition. PRIME reveals experimentally validated biophysical determinants of TCR recognition and also establishes correlations with T cell potency. MixMHCpred is integrated for predictions of antigen presentation and TCR recognition. Beside the above-mentioned features, it improves the overall prioritization of neoepitopes. Another notable prioritization algorithm is DeepImmuno (210), a CNN based tool that predicts the epitope immunogenicity for CD8+ cells of 9-10-mer peptides. The prediction can run from the command line or from their web interface. The easy-to-use web interface has MHCflurry integrated to not only predict the immunogenicity of the specific HLA-peptide pairs, but the binding affinity score as well. DeepImmuno includes an independent generative adversarial network model, which can generate immunogenic peptide with the possibility of training your own model.

Most of the tools can predict neoepitopes from SNVs, some also incorporate INDELs (pVACseq (211), MuPeXI (207), TSNAD (212), CloudNeo (213), Epidisco (214), pTuneos (215), antigen.garnish (195), NeoPredPipe (216), NeoEpiScope (217), OpenVax (218)). A few focus solely on INDELs (ScanNeo (219)) or gene fusions (NeoFuse (220), INTEGRATE-neo (221)), while others allow users to input the variants as peptides (EDGE (97), DeepHLApan (103)).

A summary of various integrated pipelines and software tools for neoantigen discovery is provided in **Table 5**.

**Table 5:** – Integrated software for neoantigen prediction and prioritization.

Intagrated software for neoantigen prediction and prioritization			
Tool name	Year	Short description	URL
**FRED2**	2016 (204)	FRamework for Epitope Detection, provides a string-of-beads poly-peptide for vaccine	http://fred-2.github.io
**MuPeXI**	2017 (207)	Mutant peptide extractor and informer, provides a list of peptides	https://services.healthtech.dtu.dk/services/MuPeXI-1.1/
**TIminer**	2017 (208)	Tumor Immunology miner, predicted neoantigen as output	https://icbi.i-med.ac.at/software/timiner/timiner.shtml
**TSNAD**	2017 (212)	Tumor-Specific Neoantigen Detector	https://github.com/jiujiezz/tsnad
**CloudNeo**	2017 (213)	Cloud pipeline, computes HLA type and neoantigens	https://github.com/TheJacksonLaboratory/CloudNeo
**INTEGRATE-neo**	2017 (221)	Gene fusion prediction and neoantigen computation from gene fusions	https://github.com/ChrisMaherLab/INTEGRATE-Neo
**Epidisco**	2017 (214)	Highly-configurable genomic pipeline supporting variant calling, epitope discovery, and vaccine generation	https://github.com/hammerlab/epidisco
**Neopepsee**	2018 (222)	Provides a rich annotation of candidate peptides with immunogenicity-related values	https://sourceforge.net/projects/neopepsee/
**pTuneous**	2019 (215)	Prioritizing SNV-based candidate neoepitopes	https://github.com/bm2-lab/pTuneos
**antigen.garnish**	2019 (195)	Open-source R package for neoantigen quality analysis	https://github.com/andrewrech/antigen.garnish
**NeoPredPipe**	2019 (216)	High-throughput neoantigen prediction and recognition potential pipeline	https://github.com/MathOnco/NeoPredPipe
**ScanNeo**	2019 (219)	Identifying INDEL-derived neoantigens using RNA-seq data	https://github.com/ylab-hi/ScanNeo
**DeepHLApan**	2019 (103)	Neoantigen prediction including HLA-peptide binding and immunogenicity	https://github.com/jiujiezz/deephlapan, http://biopharm.zju.edu.cn/deephlapan
**NeoFuse**	2020 (220)	Predicting fusion neoantigens from RNA sequencing data	https://icbi.i-med.ac.at/software/NeoFuse/NeoFuse.shtml
**Neoepiscope**	2020 (217)	Uses assembled haplotype output of HapCUT2 to enumerate neoepitopes arising from more than one somatic mutation	https://github.com/pdxgx/neoepiscope
**OpenVax**	2020 (218)	Identifying somatic variants, predicting neoantigens, and selecting the contents of personalized cancer vaccines	https://github.com/openvax/neoantigen-vaccine-pipeline
**pVACtools**	2020 (206)	Prioritizing neoantigens from VCF, FASTA file, resulting from gene fusions, generate DNA-vector neoantigen sequence	http://www.pvactools.org
**INeo-Epp**	2020 (223)	Random forest classifier for T cell immunogenic HLA-I presenting antigen epitopes and neoantigens	http://www.biostatistics.online/ineo-epp/neoantigen.php
**neoANT-HILL**	2020 (224)	Toolkit for the identification of potential neoantigens	https://github.com/neoanthill/neoANT-HILL
**DeepAntigen**	2020 (225)	Neoantigen prioritization based on 3D genome information and deep sparse learning	https://yishi.sjtu.edu.cn/deepAntigen/
**TruNeo**	2020 (226)	Predicts neoantigens based on multiple biological factors such as peptide-MHC binding, proteasomal cleavage and TAP transport efficiency predictions	https://github.com/yucebio/TruNeo
**NeoFox**	2021 (227)	A tool that provides a comprehensive description of neoantigen candidates by proposed features. Annotate neoantigen candidates with 16 neoantigen features.	https://github.com/TRON-Bioinformatics/neofox
**TSNAD v2.0**	2021 (228)	Tumor-Specific Neoantigen Detector, providing neoantigens	https://github.com/jiujiezz/tsnad, http://biopharm.zju.edu.cn/tsnad/
**PRIME**	2021 (209)	Predictor of immunogenic epitopes, prioritization pipeline	http://prime.gfellerlab.org/, https://github.com/GfellerLab/PRIME
**DeepImmuno**	2021 (210)	DL-empowered prediction of immunogenic peptides	https://github.com/frankligy/DeepImmuno
**ProGeo-Neo v2.0**	2022 (229)	Mining tumor specific antigens from WGS/WES genomic and RNA-seq data, verifying peptide-MHCs by MaxQuant with mass spectrometry proteomics data searched against customized protein database	https://github.com/kbvstmd/ProGeo-neo2.0
**Seq2Neo**	2022 (230)	Pipeline for cancer neoantigen immunogenicity prediction	https://github.com/XSLiuLab/Seq2Neo
**PGNneo**	2023 (231)	Proteogenomics-Based Neoantigen prediction Pipeline in Noncoding Regions	https://github.com/tanxiaoxiu/PGNneo
**LENS**	2023 (232)	Neoantigen prediction based on SNVs, INDELs, fusion events, splice variants, cancer-testis antigens, overexpressed self-antigens	https://gitlab.com/landscape-of-effective-neoantigens-software
**GeNeo**	2023 (233)	Toolbox on Galaxy server maintained at the University of Connecticut	https://neo.engr.uconn.edu/

## 8 Tumor neoantigen data collection

The training of novel and improved algorithms requires continuous accumulation of verified tumor neoantigen data. Several studies have curated cancer antigen data, and constructed publicly available cancer antigen resources. These databases support the community in understanding the landscape of antigen presentation and provide necessary information for the development of neoantigen prediction tools. In addition to the well-curated data sets, several so-called *in silico* neoantigen databases that omit the experimental validation step have been built by taking advantage of existing neoantigen prediction software.

There are several well-curated datasets. One of the widely used, well-known resource is the Immune Epitope Database and Analysis Resource (IEDB) (88). It is a freely available comprehensive repository for diverse immunological data. This database contains experimental data from various host organisms about peptidic and non-peptidic epitopes, MHC ligand (Class I and II), T cell and B cell assays with a chance to gain insight into the possible disease context such as allergy, autoimmune or infectious diseases (234, 235). The database exists since 2003 and due to its enormous data content with over 1,600,000 epitopes and availability, this database is integrated in many other databases we have mentioned. However, IEDB’s data sets of verified T cell epitopes primarily consists of epitopes from bacteria or viruses and were not obtained by standardized experimental methodologies in the context of cancer. Furthermore, CEDAR (236) is the cancer epitope focused companion site of IEDB. This freely available database is similarily built to its companion and houses over 1,290,000 epitopes. Here, B cell, T cell and MHC ligand assay results are available in various hosts focusing on cancer types and stages.

Further curated databases include NeoPeptide (237), dbPepNeo (238), dbPepNeo 2.0 (239), TANTIGEN (240) and NEPdb (241). NeoPeptide focuses on cataloguing neoantigens from somatic mutations across different cancer types from clinical trials and in vitro experiments. At the time of its creation in 2019 it already contained 36,000 antigens and over 180,000 epitopes which has been expanded since (10). It provides details on various neoantigen characteristic such as mutation site, sequence and MHC restriction. The dbPepNeo databases include curated information about neoantigen data validated by mass spectrometry or immunoassays in human tumors. While version 1 focuses on validated MHC-I antigens in various tumor types, in version 2 the included neoepitope candidates increased to over 840,000 while also adding MHC-II data. Both versions help the user by categorizing all neoantigen’s confidence based on the strength of the experimental validation. TANTIGEN focuses on cancer antigens whose HLA binding is experimentally validated from tumor tissues. Over 1,000 tumor peptides from close to 300 proteins are catalogued based on which the T cell epitopes and HLA ligands are easy-to-list. However, it does not include peptides shown to be ineffective and lacks any association with clinical data. NEPdb was constructed via curating published literature with a semi-automatic pipeline by parsing and filtering abstracts with NLP toolkit. It includes curated data of 173 MHC-I and MHC-II neoepitopes and over 17,000 non-immunogenic peptides from 23 tumor types. The validation focuses both on *in vitro* and *in vivo* T cell assays.

Also, there are databases on verified binding and presentation. This category includes caAtlas (242), SPENCER (243), IEAtlas (244), HLA Ligand Atlas (245) and CARMEN (246). caAtlas is a database that contains information about mass spectrometry results of 9 cancer types and non-tumor samples. The data focuses both on MHC-I and MHC-II molecules and comprises around 140,000 modified peptides. SPENCER focuses on small peptides in cancer patients that are encoded by non-coding RNAs. The database contains mass spectrometry data of 15 cancer types from over 1,700 patients resulting in the identification of near 30,000 small peptides encoded by non-coding RNA in tumors. IEAtlas collects the immunopeptidome data of mass spectrometry datasets to find epitopes that bind MHC-I/II from non-coding regions. Currently over 245,000 such epitopes are identified from 15 tumor types and 30 non-tumor tissues. the database HLA Ligand Atlas provides a collection of natural HLA ligands presented on benign tissues. Natural HLA ligand information could be important for further tool development.

Besides the experimentally verified databases there are also a number of *in silico* predicted neoantigen databases with an enormous variety of potential neoantigens. TSNAdb v1 (247) collected information about millions of potential neoantigens from somatic mutation data. The predictions of version 1.0 are based on the HLA data of 16 tumor types collected from TCGA (68) and TCIA (248) and are generated by NetMHCpan. TSNAdb v2.0 (249) upgrades its toolkit to use DeepHLApan, MHCflurry and NetMHCpan and predicted neoantigens not only from SNVs but from INDELs and fusions. The altered criteria in v2.0 decreased the false-positive predictions resulting in almost 400,000 SNV-derived, around 140,000 INDEL derived and over 11,000 fusion-derived predicted neoantigens. TSNAdb includes HLA binding info for both mutant and wild-type peptides thus, facilitating the assessment of the DAI (247). TRON Cell Line Portal (TCLP) (250) catalogues MHC types and predicted neoepitopes amongst other publicly available data of 1,082 cancer cell lines. The data focuses on both MHC-I/II neoantigens in a cell-line-specific manner.

The set of verified neo-epitopes is still limited, and we envisage that larger neo-epitope datasets will lead to additional refinements in immunogenicity predictions. For a summarized overview of the above-mentioned neoantigen databases, see **Table 6**, for a summary on immunology related databases and datasets see, **Table 7**.

**Table 6:** – Neoantigen databases.

Neoantigen databases			
Database name	Year	Short description	URL
**TSNAdb**	2018 (247)	Predicted and validated neoantigens based on pan-cancer immunogenomics analyses	https://pgx.zju.edu.cn/tsnadb1/
**NeoPeptide**	2019 (237)	Catalog of epitopes derived from neoantigens captured from literatures and immunological resources	https://github.com/lyotvincent/NeoPeptide
**dbPepNeo**	2020 (238)	Collection of experimentally validated neoantigens	http://www.biostatistics.online/dbPepNeo/
**NEPdb**	2021 (241)	T cell Experimentally-Validated Neoantigens and Pan-Cancer Predicted Neoepitopes	http://nep.whu.edu.cn/
**TANTIGEN 2.0**	2021 (240)	Database of T cell epitopes and HLA ligands	http://projects.met-hilab.org/tadb
**HLA ligand atlas**	2021 (245)	Benign reference of HLA-presented peptides	https://hla-ligand-atlas.org
**caAtlas**	2021 (242)	An immunopeptidome atlas of human cancer	http://www.zhang-lab.org/caatlas/
**dbPepNeo2.0**	2022 (239)	Database for Human Tumor Neoantigen Peptides from Mass Spectrometry and TCR Recognition	http://www.biostatistics.online/dbPepNeo2
**TSNAdb v2.0**	2022 (249)	Predicted and validated tumor-specific neoantigen database	https://pgx.zju.edu.cn/tsnadb
**CAD**	2022 (251)	Cancer Antigens Database	http://cad.bio-it.cn/
**SPENCER**	2022 (243)	Database for small peptides encoded by noncoding RNAs	http://spencer.renlab.org
**IEAtlas**	2023 (244)	Atlas of HLA-presented immune epitopes derived from non-coding regions	http://bio-bigdata.hrbmu.edu.cn/IEAtlas
**CARMEN**	2023 (246)	Database generated from 80 different immunopeptidomics mass spectrometry datasets collected between 2015-2022	Not available
**CEDAR**	2023 (236)	Cancer Epitope Database and Analysis Resource	https://cedar.iedb.org/
**Neodb**	2023 (252)	The webserver contains neoantigen prediction tools; curated, experimentally validated immunogenic neoantigen dataset; Driver mutation derived potential neoantigens; immunogenicity prediction tool	https://liuxslab.com/Neodb/

**Table 7:** – Immunology-related databases and datasets.

Immunology-related databases and datasets			
Database name	Year	Short description	URL
**IMGT**	2015 (253)	International Immunogenetics Information System	https://www.ebi.ac.uk/ipd/imgt/hla/index.html
**TCLP**	2015 (250)	TRON Cell Line Portal	http://celllines.tron-mainz.de
**MIRA**	2015 (175)	Antigen-Specific T cell Receptors	https://github.com/mnielLab/NetTCR-2.0/tree/main/data
**McPAS-TCR**	2017 (172)	Manually curated catalogue of pathology-associated TCR sequences	http://friedmanlab.weizmann.ac.il/McPAS-TCR/
**TCIA**	2017 (254)	Cancer Immunome Atlas, links tumor genotypes with immunophenotypes, providing an index for immunotherapy response	https://tcia.at/home
**SysteMHC Atlas**	2018 (255)	Data Repository for Immunopeptidomic Analyses	https://systemhcatlas.org
**VDJdb**	2018 (256)	Database of T cell receptor sequences with known antigen specificity	https://vdjdb.cdr3.net/
**IEDB**	2019 (88)	Immune Epitope Database	https://www.iedb.org
**TBAdb, PIRD**	2020 (174)	Pan immune repertoire database	https://db.cngb.org/pird/
**TCRdb**	2021 (147)	Database for T cell receptor sequences with powerful search function	http://bioinfo.life.hust.edu.cn/TCRdb
**UcTCRdb**	2023 (257)	T cell receptor sequence database with online analysis functions	http://uctcrdb.cn/

## 9 Benchmark for neoantigen prediction

In 2016, the Tumor Neoantigen Selection Alliance (TESLA) was established as a collaborative effort to identify the most effective predictive algorithms for targeting neoantigens through large scale validation. Supported by the Parker Institute for Cancer Immunotherapy and the Cancer Research Institute (CRI) (189, 258), TESLA involved 35 public and private research teams worldwide. Each team employed its own unique neoantigen prediction algorithms to identify and prioritize neoantigens. The initial focus was on advanced melanoma, colorectal cancer and non-small cell lung cancer (NSCLC). Genomic data from the same six patient samples (3 melanoma, 3 NSCLC) was provided by the Alliance. The immunogenicity of candidate neoantigens was validated through MHC-restricted T cells in subject-matched peripheral blood mononuclear cells (PBMC). This study highlighted the significant differences in the prediction methodologies among the groups. No single methodology identified every neoantigen, nor a large majority of neoantigens, indicating the need for a standardized approach.

Besides testing the already existing predicting algorithms, the other goal of the TESLA was to identify key parameters shaping tumor epitope immunogenicity. The Alliance determined that approximately 50% of immunogenic epitopes are characterized by strong MHC binding affinity, prolonged half-life, high expression, and either low agretopicity or high foreignness. A model based on these five peptide features associated with presentation and recognition was developed and tested against independent cohorts of cancer samples. TESLA data is available (259) to qualified investigators and provides opportunities to benchmark the performance of neoantigen workflows.

Using the TESLA dataset, Buckley et al. (260) evaluated performance of seven publicly available methods - IEDB model (261), NetTepi (262), iPred (263), Repitope (264), PRIME (209), DeepImmuno (210) and Gao (265) - predicting whether an MHC-presented peptide might invoke a T cell response (i.e. whether a peptide is immunogenic). Filtering the TESLA dataset, originally comprising cancer peptides from 13 class I alleles, to retain alleles for which all models are applicable, and excluding peptides observed in any model’s training data, resulted in 27 immunogenic and 372 non-immunogenic peptides (lengths 9 or 10 aminoacids) that were experimentally tested against seven HLAs. They observed high numbers of false positives for all model. In this benchmark, PRIME identified 26 neoantigen from the total 27, successfully reaching the highest number of identified TESLA neoantigens.

## 10 Challenges and potential solutions to gain widespread adoption of AI applications for neoantigens discovery

**Figure 4:**
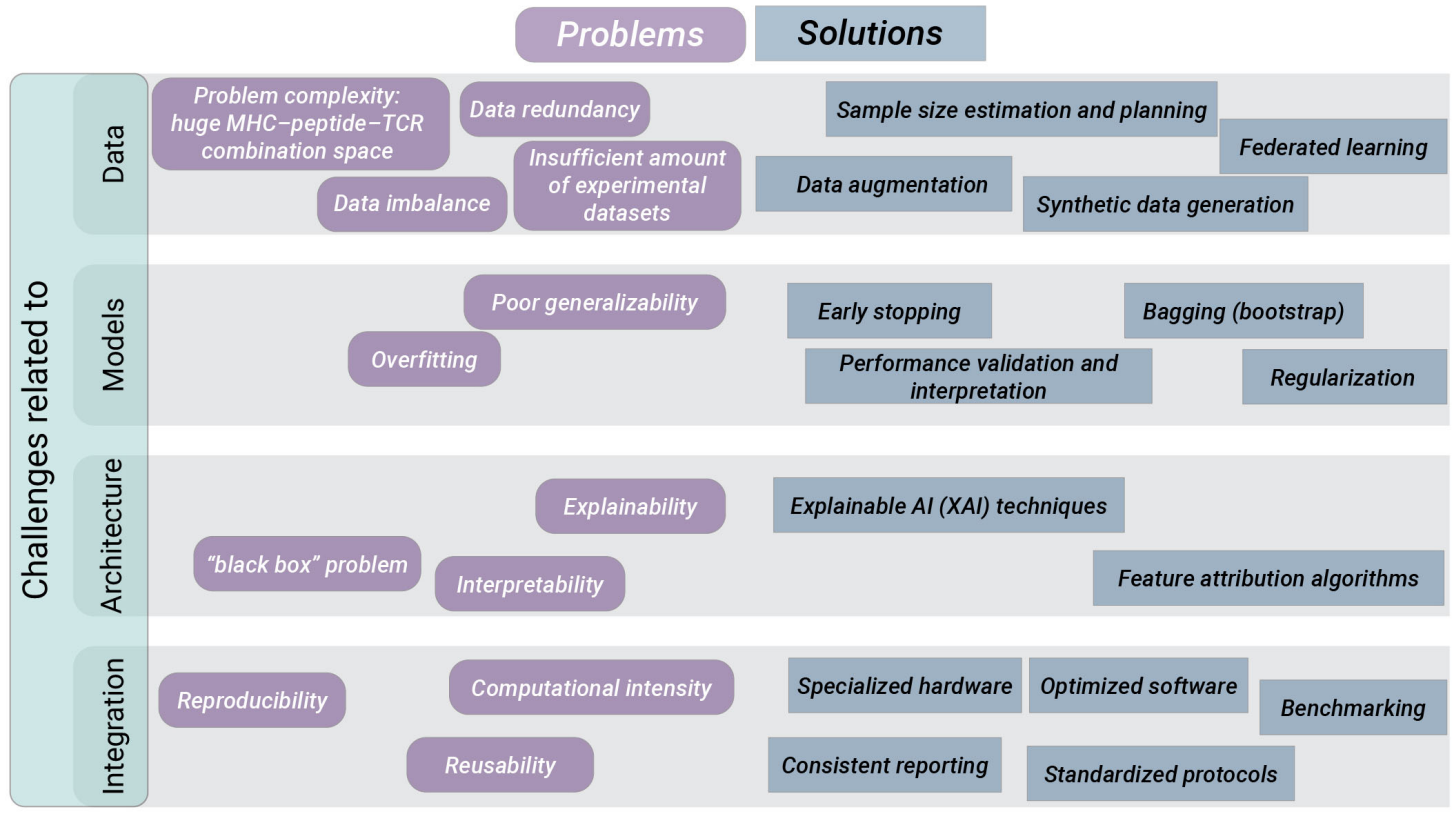
Challenges and potential solutions to promote widespread clinical use of AI applications for neoantigens discovery. We distinguish challenges that must be addressed for successful AI integration into clinical praxis as related to data, models, AI architecture and technical integration. For each group of challenges we list various algorithmic, experimental and organizational approaches carrying the potential to overcome the respective challenges.

Learning from a large set of data and identifying patterns of interest is the greatest strength of AI. The integration of AI applications in cancer immunotherapy and personalized medicine holds great promise, however, also comes with various technical and implementation challenges. **Figure 4** summarizes the introduced bottlenecks of AI-based neoantigens discovery along with their potential solutions.

### 10.1 Challenges related to data

#### 10.1.1 Insufficient amount of available well-curated data

Data scarceness, data accuracy, and problem complexity contribute to challenges with models training. Available experimental datasets are limited in volume, diversity and standardization. Additionally, there is a lack of experimental data of binding affinity and antigen presentation for many HLA alleles. Furthermore, for many datasets consistent biological definitions are not considered or differ between studies, e.g. distinguishing between pre-existing and *de novo* T cell responses upon neoantigen vaccination.

Problem complexity is imposed by the huge MHC–peptide–TCR combination space, the length variations of TCRs, and inter- and intra-patient variability of TCRs or MHCs. Running AI training procedures on a limited or disparate data may result in overfitting and biased outcomes, compromising the reliability of future predictions.

#### 10.1.2 The lack of experimentally verified negative data and the issue of data imbalance

EL/MS experimental approach reports only the presence of a peptide at the cell’s surface, but cannot identify the absence of a peptide from the individuals’ immunopeptidome. The prediction of peptide-MHC binding is a quintessential classification problem. For binary classification, there should be a sufficient number of observations in both positive and negative classes. Otherwise, the imbalance will lead to a bias of the classifier trained on these data and therefore, the creation of artificial negative examples (decoys) is required. However, insufficient consideration of the source of the negative examples can lead to further biases (266). Recently a homology-based method Neglog was proposed (267) to infer more negative data from very limited experimentally verified Negatome (i.e., pairs of proteins that do not interact). Neglog outperformed pure random sampling, and independent test on negative data is indispensable for bias control, which is usually neglected by existing studies (267). Negative data sampling also needs to be properly addressed for computational prediction of peptide-MHC and TCR–peptide binding.

#### 10.1.3 The influence of dataset homology

Another problem is data similarity. Datasets contain many epitopes that are either identical or very similar to each other, which results in data redundancy. If not properly managed, redundancy can lead to overfitting. By performing homology reduction procedures, some of the tools take redundancy into account. The influence of dataset homology on protein secondary structure prediction was investigated by Chen et al. (268), and a rigorous evaluation strategy was proposed.

#### 10.1.4 The lack of sample size determination

How much training data is required for AI application? The minimum dataset size required for effective training of AI models remains unclear in the biomedical sector. The rule “the more data, the better” is not realistic in the biomedical sector which faces technological limitations in acquiring data. Theoretical investigations concerning sample size planning for classification models (269) and sample size estimation for effective modelling of classification problems (270) are available and should be contemplated.

#### 10.1.5 Algorithmic and model-driven solutions to data challenges

There are approaches in the biomedical and general domain aiming to balance the dataset used for AI training. Data reweighting helps to compensate under-represented subgroups by duplicating the minority class data. Data perturbation increases the diversity of the dataset by adding “noise” to existing samples. Data augmentation is a process of generating synthetic data exploiting algorithms such as generative adversarial networks (GANs). GANs consist of two main components trained simultaneously using adversarial training: a generator model generating samples similar to real data, and the discriminator model attempting to distinguish between real and generated samples. We already mentioned DeepImmuno (210) using GANs to generate immunogenic peptides. Federated learning is another approach to work with limited data sources or skewed distribution in the dataset. In federated learning, a central machine aggregates learning from other devices referred to as clients, collaboratively training a model while ensuring that their data remains decentralized. The idea to generate a global model via exchanging parameters (e.g. the weights and biases of a deep neural network) between the local nodes without explicitly exchanging data samples was motivated by the issues such as data privacy and data access rights.

### 10.2 Challenges related to models

#### 10.2.1 The problem of overfitting and lack of generalizability

Memorizing the training examples without learning any generalizable patterns by the model is a problem called overfitting. If a predictor overfits to the training data, its actual prediction accuracy on a new data will be worse than the one reported (271). Increasing the complexity of AI model (e.g. increasing the number of layers of ANN and thus the number of parameters) can result in overfitting and consequently in poor generalizability of the model. To address this issue, various methods can be employed. *Early stopping* technique prevents overfitting by stopping the training process at the moment the test error starts to increase. Resampling methods such as Bagging or Bootstrap, in particular the optimism-adjusted bootstrap (OAD) (272), aim to increase the generalization capability of the model by training multiple base learners on randomly sampled portions of data and then aggregating the learners. Regularization improves the model’s generalization capability by setting the weights of features in the model closer to zero, reducing the influence of insignificant features. *Dropout* is a kind of regularization technique employed in deep learning, working by randomly dropping neurons out of the network during the training with the aim to prevent any neuron from becoming too influential. Cross-validation divides the dataset into multiple equal parts and evaluates the model’s performance by using each segment as a test set in turn. Performance validation and interpretation, identification and correction of biases, are essential for more reliable, accurate, and generalizable AI models.

#### 10.2.2 Performance metrics demonstrating the quality of a model are not standardized

To assess the prediction performance of AI algorithms, numerous performance metrices are alternatively used. These include accuracy (Acc), sensitivity (Sn), specificity (Sp), F1 score, the Matthews Correlation Coefficient (MCC), the area under the receiver operating characteristic (ROC) curve (AUC), and Positive Predictive Value (PPV). The findings of *in silico* studies are presented in a heterogeneous manner and are difficult to compare. The suitability of performance metrics may also depend on the data situation at hand. For example, when diagnosing classification model performance on highly imbalanced datasets, ROC-AUC can underrepresent the minority class and be therefore misleading, while precision–recall area under the curve (PR-AUC), which summarizes model precision and recall, represents the balance of classes within the testing dataset more accurately (273).

### 10.3 The challenge of interpretability: AI models operate as a “black box”

*“Has artificial intelligence become alchemy?”* (274) Another important obstacle experienced by AI applications is the lack of understanding the methodology and the human inability in explaining the precise steps leading to predictions. How the models make the predictions and what the models learn from the input data remains largely unknown. The AI is in its golden era and the advances and possibilities are almost endless. However, to trust model predictions completely, it is vital to understand the processes that transforms inputs into outputs. There have been several attempts to improve the interpretability of ML models. Vig et al. (275) used the transformers attention mechanism to show that some of the transformer’s nodes were able to learn biological properties of proteins (e.g. secondary structure, binding sites etc.).

In the context of peptide presentation by MHC class I proteins it will be important to identify the most influential parts of the input amino acid sequences contributing to the output. To tackle this challenge, the authors of ImmunoBERT (85) presented application of two interpretability techniques developed in the field of computer vision, SHapley Additive exPlanations (SHAP) (276) and Local Interpretable Model-agnostic Explanations (LIME) (277), for interpreting BERT architecture predictions. Using the tool Captum (278), one can apply a wide range of feature attribution algorithms to attribute the predictions of a DL-based image classifier to their corresponding image features. Adoption of such algorithms to the analysis of sequence information would provide new insights in the field.

### 10.4 Difficulty in integration of AI applications

#### 10.4.1 Benchmarking the different AI or ML tools

AI or ML tools are excessively difficult to benchmark in the clinical setting despite the fact that they can be trained with existing databases on patient data. One clinical study with a prediction tool cannot be directly compared to another clinical study that uses another tool, since the patients and the neoantigens are different.

#### 10.4.2 Reproducibility and reusability of AI models

To improve transparency and reproducibility, guidelines have been established for developing and reporting ML predictive models in biomedical research (279). These guidelines promote consistent reporting of model specifications, including potential limitations of the model such as assumed input and output data format, pitfalls in interpreting the model, potential bias of the data used in modeling, generalizability of the data. In addition, sharing of well documented code for the model together with transparent descriptions of the optimized hyperparameters and hardware specifications is another aspect that would ensure that AI algorithms are transparent and reproducible. Collaborative initiatives for generation of joint guidelines and consensus recommendations, as well as translation them into standardized protocols will play a crucial role in driving the widespread adoption of AI-based solutions.

#### 10.4.3 AI is computationally intensive

Successful application of AI requires proper computational infrastructure, including specialized hardware such as graphics processing units (GPUs), as well as optimized software for reduced computational needs (e.g. Q SLAM Technology), and solutions for integrated management of data and resources.

### 10.5 The ethical and legal implications of using AI

Algorithms do not accept responsibility or legal liability for their decisions and errors. Careful development, testing, and evaluation is required before integrating AI systems for patient care (280, 281). These challenges must be addressed to fully harness the potential of AI in cancer immunotherapy and personalized medicine.

## 11 Discussion

AI has already proven to be useful in everyday life from refining the text of manuscripts to troubleshooting codes (282). However, the risks are higher when applying AI to human health. The implementation of AI in general clinical practice can be a sensitive topic. Medical professionals spend decades learning, practicing, improving and the gained experience along the way is extremely valuable. Comparing AI that has unknown or unexplainable processes to the medical professional when it comes to diagnosis and decision making related to possible therapy or necessary surgery, is a rather delicate topic for discussion (283).

Nonetheless, it is undeniable that AI technology is currently needed in the medical field. One such field where AI´s involvement is certainly required is cancer immunotherapies. In the past decades, immunotherapy has become increasingly important as a new form of cancer therapy. For the development of cancer vaccines, quick and efficient processing of large data is required. One challenge is to identify tumor-specific antigens, the majority of which are unique for individual patients. Combining tumor sequencing data with the use of predictive algorithms based on machine learning and artificial intelligence allows clinical investigators to accelerate identification of therapeutically relevant neoantigens.

We reviewed multiple tools and a broad selection of prediction servers for neoantigen detection based on advanced AI methodologies. These tools are still far from widespread use in clinical practice as it can be difficult for users to choose the best server. There is a lack of reference data that should serve as an open benchmark to compare the approaches and validate the concordance of predictions among different tools. We encourage the standardization of techniques and harmonized protocols for sequencing, mutation detection, immunogenicity testing, and neoantigen candidate prioritization.

Our work highlights the barriers of applicability and clinical adoption of AI approaches. The insufficiency of experimental data for training and associated with it the lack of generalizability of AI-based models represents the major challenge. Novel approaches capable to overcome the critical role of data limitations are required for further development of in silico methods. Transfer learning has become increasingly relevant in this regard. AI models that can efficiently use all of the limited available data and transfer knowledge from other sources are extremely valuable.

Carefulness must be applied to the issue of performance guarantees both for training the model and for assessing how it will perform when deployed. Standard statistical and ML methods should be employed, such as bootstrap or a Bayesian method to assess prediction confidence intervals, to quantify the uncertainty of AI model in the output, and analyzing the sensitivity of the model’s output to certain parameters. Often the target and loss function used for training may not match the target and loss function important for the users. Bridging this training-application gap can be addressed by grounding methods, i.e. supplementing the model’s training with context-specific information, improving its ability to function effectively in disparate real-life situations.

A mechanistic explanation of the relationship between the peptide sequence, HLA allele and binding affinity remains an open topic of investigation. AI-based tools provide a potential solution in two ways: 1) Deep learning approaches can learn features automatically from unstructured data, bypassing the need to discover a mechanistic explanation. 2) Explainable AI techniques, such as attention mechanism, may be able to provide clues about aspects of the relationship that require further investigation. The two possibilities are not mutually exclusive and if early efforts focus on producing accurate and generalizable black-box models, then later efforts should attempt to use explainable AI techniques to understand the reasoning the model uses to make its predictions. As we navigate the path forward in personalized cancer immunotherapy, several questions remain. How can we expand the collection of well-curated neoantigen data, particularly for rare cancer types? What additional factors beyond peptide properties, such as protein structure and post-translational modifications, should be considered for neoantigen prediction? How can we enhance the interpretability of AI models, making them more transparent and accountable? These questions, among others, represent exciting avenues for future research and innovation.

By depositing the results of experiments and clinical trials in public databases, investigators will assist in making neoantigen prediction models more generalizable. Companies should agree to mutually exchange information beneficial to all parties in a benchmarking group and share the results within the group. As clinical studies will continually evolve to become more inclusive, harmonized and easily accessible, the aforementioned challenges of clinical integration of AI will also be bridged.

This review focuses specifically on AI and neoantigens, however, the use of AI approaches to predict cancer immunotherapy efficacy (284) and patient’s response to immunotherapy (285) is also worth mentioning. AI can utilize complex images such as histopathological slides and follow-up CT scans, extract information from multi-omics data (genomics, transcriptomics, epigenomics, proteomics, radiomics), integrating it with clinical data (medical history, laboratory tests, demographic information) to distinguish immunotherapy responders from non-responders. One of the major challenges in immunotherapy is to determine which patients are likely to benefit from the therapy. Tumor mutational burden (TMB) was proposed as biomarker and approved by the FDA to select patients eligible to receive pembrolizumab. The review of Addala et al. (285) discusses cancer-intrinsic and cancer-extrinsic features that can be analysed. Besides TMB, genomic intratumor heterogeneity (ITH) can also be used as cancer-intrinsic feature for outcome prediction, as it was linked to treatment resistance, recurrence and reduced patient survival. Advances in single-cell analysis technologies enable further insights into genomic ITH, neoantigen formation and presentation at single-cell level. Cancer-extrinsic features encompass the cellular composition of the tumor microenvironment (TME). AI deconvolution tools, e.g. CIBERSORTx (286), provide estimates of the immune cell proportions in the TME. The complex model capable to integrate multiple factors including tumor purity, TME composition, tumor evolution, genomic ITH and immunogenic neoantigen load would be of great importance. The parameters that govern the immunogenicity still remain largely unknown. The review of Xie et al. (287) outlines further barriers that must be overcome to enable effective anti-cancer immunotherapies. Tumors can escape from immunological surveillance through a number of mechanisms, including the loss of neoantigens induced e.g. by transcriptional repression or epigenetic silencing, disruption of neoantigen peptides presentation, and immunosuppressive TME. To compensate for the loss of targetable neoantigens, personalized neoantigen-specific immunotherapy should target multiple neoantigens (288). In the work of Xie et al. (287) additional compensatory strategies to address the issue of immune evasion of tumor cells are discussed.

The recent publication of Donisi et al. (289) also considers the mechanisms behind the resistance to immune therapeutic agents, in particular, the tumor immune microenvironment (TIME), a part of the TME, or microbiome influencing immune cells in the TME etc., and reviews multi-omics and AI approaches, e.g. those for dissecting the TME or inferring novel microbiome-linked biomarkers (289).

In conclusion, the field of neoantigen prediction is at the forefront of personalized cancer immunotherapy. The collaborative efforts of researchers, computational biologists, and immunologists have brought us closer to harnessing the full potential of neoantigens for precision medicine. With continued advancements in software, databases, and AI, we are on the cusp of a new era in cancer treatment, one that holds the promise of tailored immunotherapies that target the unique molecular signatures of each patient’s tumor. As both academic and industrial endeavors keep on to tackle the challenges outlined in this article, the future of personalized cancer immunotherapy appears brighter than ever.

## Author contributions

AB: Conceptualization, Investigation, Writing – original draft, Writing – review & editing. ZN: Conceptualization, Investigation, Visualization, Writing – original draft, Writing – review & editing. FL: Writing – review & editing. MB: Writing – review & editing. MM: Writing – review & editing. MD: Funding acquisition, Writing – review & editing. LC: Writing – review & editing. RK: Conceptualization, Funding acquisition, Supervision, Writing – original draft, Writing – review & editing.

## Footnotes

1 Radford A, Narasimhan K, Salimans T, Sutskever I. Improving Language Understanding. (2018) 4. OpenAI.com.
